# Spatiotemporal Trends in Stunting Prevalence Among Children Aged Two Years Old in Rwanda (2020–2024): A Retrospective Analysis

**DOI:** 10.3390/nu17172808

**Published:** 2025-08-29

**Authors:** Seleman Ntawuyirushintege, Ayman Ahmed, Georges Bucyibaruta, Emmanuel Edwar Siddig, Eric Remera, Fabrizio Tediosi, Kaspar Wyss

**Affiliations:** 1 College of Medicine and Health Sciences, University of Rwanda, Kigali 4285, Rwanda; 2 Swiss Tropical and Public Health Institute (Swiss TPH), 4123 Allschwil, Switzerland; 3 Faculty of Medicine, University of Basel, Petersplatz 1, 4001 Basel, Switzerland; 4 Pan-Africa One Health Institute (PAOHI), Kigali 11KG ST203, Rwanda; ayman.ame.ahmed@gmail.com; 5 Institute of Endemic Diseases, University of Khartoum, Khartoum 11111, Sudan; 6 Center for Equity in Global Surgery, University of Global Health Equity, Kigali 4285, Rwanda; 7 Faculty of Medical Laboratory Sciences, University of Khartoum, Khartoum 11111, Sudan; emanwelleds389@gmail.com; 8 Division of Research Innovation and Data Science, Rwanda Biomedical Center, Kigali 4285, Rwanda

**Keywords:** child growth and development, child malnutrition, food security, community screening, community engagement, Rwanda, program monitoring and evaluation, climate change, Africa

## Abstract

**Background and Objective:** Stunting remains a critical public health concern affecting child growth and development, particularly among children under two years of age in low- and middle-income countries, including Rwanda. This study investigates spatiotemporal trends in stunting prevalence from 2020 to 2024 at the sector level using national surveillance data. **Methods:** To capture regional disparities and temporal trends, we used hierarchical Bayesian spatiotemporal models, which accounted for spatial structure, temporal correlations, and interactions, to estimate stunting prevalence across districts and sectors over time. **Results:** Between 2020 and 2024, the national prevalence of stunting among children under two years decreased from 33.1% to 21.7%, representing a 34.4% change. Three districts, Kamonyi, Nyarugenge, and Ngoma, achieved reductions of >70%, whereas Rubavu, Nyabihu, and Nyaruguru saw minimal change (14–15%). By 2024, several sectors in Kicukiro, Nyanza, Nyarugenge, and Kirehe had reduced levels of stunting below the national target of 19%. **Conclusions:** Despite considerable gains, significant geographical differences persist in the stunting prevalence of children under two, underscoring the need for targeted, decentralized interventions to mitigate and eliminate this in lagging areas.

## 1. Introduction

Stunting poses significant risks to both child growth and overall development, with implications that extend from early life up to adulthood. In the shorter term, it is associated with higher morbidity and mortality, cognitive impairment, developmental delays, and increased costs due to medical interventions [1]. The longer-term issues are even greater, as it affects individuals by reducing adult stature, affects reproductive health, education, and cognitive capacities, and lowers productivity levels. Together, these outcomes contribute heavily to a reduced public health development index, as observed in Low- and Middle-Income Countries (LMICs), emphasizing the persistent challenges caused by stunting. For example, stunted children experience heightened morbidity and mortality rates, as well as cognitive deficits, motor skill challenges, and inflated healthcare costs due to necessary medical interventions [2]. In the long term, the effects are even more grave: children affected by stunting may grow into adults with reduced height, compromised reproductive health, hindered academic achievement, diminished learning abilities, and decreased productivity in the workforce. Furthermore, one of the critical repercussions of these interrelated health issues is a low public health development index, which remains a concern in regions such as Papua, evidencing the persistent challenges posed by stunting [3]. A widely accepted standard observes that a child is classified as stunted if their height falls below −2 standard deviations (SD) from the median established by the World Health Organization (WHO) Child Growth Standards for their specific age and gender. Furthermore, if a child’s height is below −3 SD from this median, they are considered severely stunted [4]. Globally, stunting among children under two years represents a major public health crisis. According to reports, approximately 45% of children who die from malnutrition fall into this age group [5]. The LMICs, particularly in Asia and Sub-Saharan Africa, continue to face this pressing health challenge [5]. Factors such as inadequate food intake, alongside prolonged exposure to recurrent infectious and respiratory diseases, have been identified as a significant barrier preventing children under two years old from recovering from stunting [6,7,8].

Growing evidence highlights the critical importance of preventing stunting, particularly during the first 1000 days of life, as this period is crucial for avoiding developmental challenges, adverse cognitive outcomes, compromised education, and future productivity challenges [6,7]. Furthermore, stunted children are at increased risk for non-communicable diseases (NCDs), obesity, and being overweight later in life [8,9]. Addressing child malnutrition requires a coordinated, decentralized approach that is both precise and multifaceted; it must not only emphasize food security but also enhance maternal and child nutrition while tackling broader social determinants that influence child health outcomes [10,11].

In Rwanda, stunting remains a critical issue. The recent Rwanda Demographic and Health Survey (RDHS) for 2019–2020 indicates that 33% and 27% of children under two years are stunted, respectively, a statistic that underscores the urgency of addressing this public health concern [11].

Rwanda committed to reducing the prevalence of stunting in children under 5 to 19% in the National Strategies for Transformation (NST1) [12,13] by 2024, to 15% by 2029 according to the Health Sector Strategic Plan (HSSPV) [11], and to 3% by Vision 2050. This ambitious goal is supported by a strategic plan that encompasses six key activities: enhancing community-based child nutrition financing and monitoring through health workers; improving antenatal care attendance; empowering nutrition centers at health facilities; increasing household incomes and food security; promoting sanitation practices; enhancing parental responsibility in child feeding, care, and overall development.

A national screening conducted by the Rwanda Biomedical Center (RBC) in November 2023 revealed that 94% of children aged 6 to 23 months received food supplements, including fortified blended foods (FBFs) and Ongera, a locally produced food supplement [14]. These initiatives are funded through government efforts and stakeholder collaboration at the district level and health center coordination. However, the persistent rates of stunting reflect ongoing challenges associated with child feeding practices, social determinants of nutrition, and co-morbidities that adversely affect nutritional outcomes [7,14].

This study aims to provide insights into trends and progress made toward reducing the prevalence and burden of stunting among the population of children under two years of age in Rwanda. To do so, we deployed spatiotemporal analysis to characterize the annual variation over recent years, as well as the geographical difference in the prevalence of stunting among the under-two population in Rwanda [15]. Understanding the spatiotemporal trends of stunting in the population in Rwanda under two years of age will inform policymakers and guide public health leaders in the strategic planning and implementation of cost-effective interventions for the prevention and control of stunting in this country.

## 2. Materials and Methods

### 2.1. Study Setting

**Figure 1:**
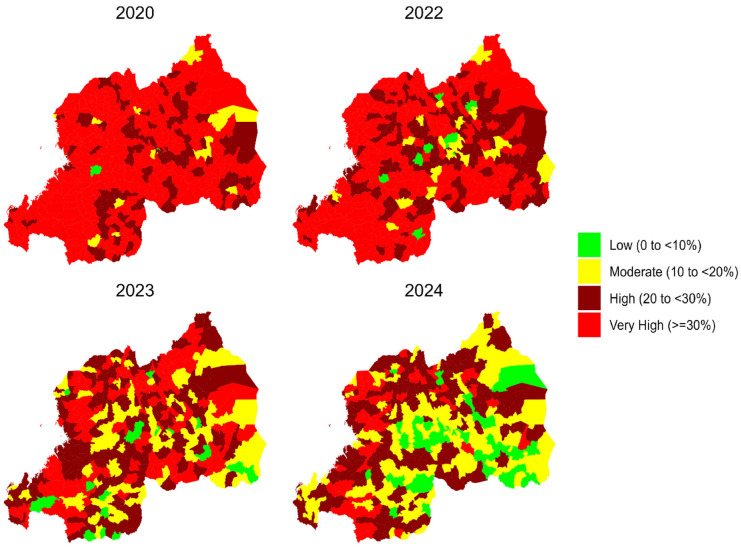
Maps of Rwanda showing the spatiotemporal changes in the prevalence of stunting among the population of children under two years old throughout the country in 2020, 2022, 2023, and 2024.

Rwanda is a landlocked country located in the eastern and central Africa region with an area of 26,338 square kilometers. It is made up of five different administrative structures. The first level is five provinces (South province, Western province, Northern province, Eastern province, and Kigali City) (Figure 1). The second level is the district level, with 30 in total. The third level is made of 416 sectors, the fourth level includes 2148 cells, and the fifth level contains 14,837 villages throughout the country.

According to the most recent national census, the country’s population is estimated to be 13,246,394 persons, with 70.3% of them being younger than 30 years old. Rwanda is characterized by a hilly landscape, and thus, it is also known as the Land of a Thousand Hills. It has a population density of 503 people per km, and an elevation between 1500 and 2000 m above sea level. Most of the country’s population, 72%, lives in rural settings.

The Ministry of Health (MoH) is the highest policy level, and through the Rwanda Biomedical Center (RBC), it coordinates the implementation of health programs, including the Maternal Child Health and Community Health (MCCH) program, in which national screening for malnutrition in children aged between 6 and 23 months is performed. The surveillance includes continuous nutrition screening for the children, indicated by anthropometric measures to identify their nutrition and growth status [14]. The Maternal and Child surveillance week is organized twice per year by the Ministry of Health (MoH) through the Rwanda Biomedical Center (RBC), and during this week, the community health workers (CHWs), under the supervision of health facilities, and local community leaders including the District Health Management Teams (DHMTs), screen children’s nutrition, immunization, and growth status. They also provide vitamin A, deworming treatment, and food supplements. Around 58,567 community health workers (four per village) are also responsible for ensuring house-to-house community education, early identification and referral of acute malnutrition to the nearest health facility, and data-driven nutrition reporting.

To ensure data quality and comparability across the sites and to control errors, before starting data collection, the CHWs completed WHO-guideline-based training and standardized field protocols for using the calibrated equipment, and repeated measurements were taken in case of implausible values for anthropometric measurements [16].

Using this routine and continuous data collection, this research aims to assess the spatiotemporal variations in stunting prevalence from 2020 to 2024, including the sector-level prevalence trend.

### 2.2. Data Source and Study Design

This study involves a retrospective analysis of data collected through the Rwanda National Maternal and Child Health (NMCH) program within the Rwanda Biomedical Center (RBC) from 2020 to 2024. The dataset includes anthropometric measurements, specifically height and weight, to determine stunting status in children under two years old. Residency information at the province, district, and sector levels was integrated with geographic boundary data obtained from the National Institute of Statistics of Rwanda to enable spatiotemporal analysis.

### 2.3. Stunting Definition and Categorization

Stunting was defined according to WHO standards as a Height-for-Age Z-score (HAZ) below −2 standard deviations from the WHO Child Growth Standards median. Districts and sectors were categorized based on the following WHO prevalence thresholds for stunting: low (<10%), moderate (10–19%), high (20–29%), and very high (≥30%) [6,17] (Table 1).

### 2.4. Sample and Data Collection

The population of this study consists of stunted children under two years old in Rwanda. Table 2 summarizes the sample per 30 districts and per year. The total sample per year was 379,381 in 2020, 299,878 in 2022, 370,752 in 2023, and 386,726 in 2024. In 2021, data were missing, as routine surveillance was suspended due to COVID-19 pandemic restrictions in Rwanda.

Data collection proceeded as follows: during the surveillance, a trained community health worker took anthropometric measurements (height, weight, Middle Upper Arm Circumference (MUAC), and age), and during the same screening, they also provided mebendazole treatment for all children as part of the national drug administration campaign to eliminate soil helminths, provide education for family planning, etc. Children who screened positive for malnutrition were referred to health facilities and communities for care. The collected data were compiled at the health center and submitted to the national MCCH program.

### 2.5. Statistical Analysis

We implemented a hierarchical Bayesian spatiotemporal disease mapping model to estimate sector-level stunting prevalence over time.

Let $y_{it}$ denote the observed count of stunted children in sector i during year t. We modeled these counts as

$$y_{it}∼Binomial(n_{it},\;p_{it})$$

where $n_{it}$ is the number of children assessed in sector i at year t, and $p_{it}$ is the probability of stunting. The log-odds of stunting were expressed as

$$logit\left(p_{it}\right)=\alpha +b_{i}+\gamma_{t}+\phi_{t}+\delta_{it}$$

where α is the overall; $b_{i}$ represents spatially structured random effects accounting for spatial autocorrelation among neighboring sectors, modeled using a reparametrized Besag–York–Mollié (BYM) conditional autoregressive prior [18,19]; $\gamma_{t}$ represents temporally structured effects modeled as a first-order random walk to capture smooth temporal trends; $\phi_{t}$ captures temporally unstructured effects modeled as independent Gaussian noise; $\delta_{it}$ represents a type I space–time interaction term capturing sector-specific deviations from temporal trends [19]. This approach allows for borrowing strength across sectors and over time, improving the precision of prevalence estimates in areas or years with sparse data.

### 2.6. Prior Selection and Model Fitting

The temporally structured effect was modeled dynamically through a nonparametric formulation for the linear predictor [20], allowing for an interaction between space and time to explain differences in the time trends of stunting across areas. The data observed consisted of counts of stunted children in each sector of Rwanda for each year from 2020 to 2024.

A Bayesian hierarchical spatiotemporal model was fitted using the Integrated Nested Laplace Approximation (INLA) framework in the R 4.4.3 software [15,20], which provides fast and accurate approximations of posterior distributions without relying on sampling-based algorithms. We applied the default prior distributions defined in INLA for model parameters, which are weakly informative and designed to ensure computational stability while avoiding overly strong prior influence. The spatially structured effect was modeled using an intrinsic conditional autoregressive (ICAR) prior, and the temporally structured effect was modeled using a first-order random walk (RW1) prior.

### 2.7. Model Fit and Convergence Assessment

Given that INLA is a deterministic approximation method, traditional MCMC convergence diagnostics do not apply. Instead, model fit was assessed using the Deviance Information Criterion (DIC), Watanabe–Akaike Information Criterion (WAIC), and Conditional Predictive Ordinate (CPO) statistics. Posterior distributions were examined for smoothness and unimodality, and sensitivity analyses with alternative priors were conducted to ensure the robustness of the findings.

### 2.8. Posterior Inference and Mapping

To facilitate the interpretation of spatial patterns, sectors were categorized according to the WHO prevalence thresholds for child stunting. This classification was applied to the model-based posterior mean prevalence estimates of sector-level stunting prevalence obtained from the fitted Bayesian spatiotemporal model. For temporal comparisons, we used the relative change in stunting prevalence as the primary measure to enable a fair comparison across sectors with different baseline prevalence levels, as it provides a scale-free metric and avoids overemphasizing changes in areas with initially high prevalence. We present maps of the posterior mean prevalence estimates, highlighting sectors where prevalence exceeds the national target threshold of 19% by 2024, classified as high or very high. These categories were used in thematic maps to illustrate spatial heterogeneity and identify sectors with elevated prevalence levels.

## 3. Results

Our analysis revealed a substantial decline (34.4% percentage change) in the national prevalence of stunting among children under two years of age in Rwanda between 2020 and 2024, decreasing from 33.1% to 21.7%. This reduction is evident in the spatial distribution maps (Figure 1), which show a shift from predominantly red areas (very high prevalence) in 2020 to more green areas (low prevalence) in 2024. Corresponding 95% credible intervals for sector-level stunting prevalence estimates were calculated and are reported in Supplementary Table S1, which lists the posterior mean and the lower and upper bounds for each sector by year (2020, 2022, 2023, and 2024). For instance, the Gahanga sector in Kicukiro district (City of Kigali) had an estimated prevalence of 23.9% in 2020 (95% CI: 21.5–26.4%) and 21.2% in 2024 (95% CI: 10.8–37.4%), reflecting relatively high prevalence. In contrast, the Cyato sector in Nyamasheke district (Western Province, rural area), depicted in red, had a very high prevalence of 67.4% in 2020 (95% CI: 64.5–70.2%) and 40.8% in 2024 (95% CI: 37.3–44.3%), highlighting persistent elevated stunting in some rural areas.

**Figure 2:**
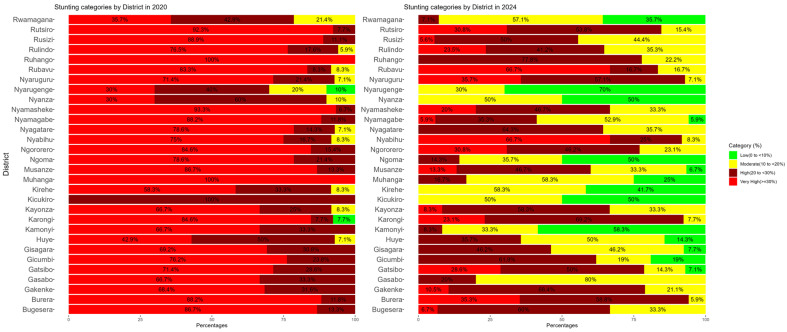
The proportional composition of stunting per the WHO cut-off among children under two years old in Rwandan districts in 2020 and 2024.

Figure 2 shows changes in the distribution of stunting prevalence categories across districts from 2020 to 2024. Each bar segment represents the percentage of sectors within a district that fall into a given stunting category. The results of these proportional changes, based on WHO cut-off values, revealed substantial progress in reducing severe stunting. Districts such as Kicukiro, Nyanza, Nyarugenge, and Kirehe have effectively eliminated severe stunting, whereas Rubavu, Nyabihu, and Burera continue to have a high or very high prevalence (≥20%) among children under two years.

Table 3 presents the estimated prevalence for each district by year, along with the corresponding absolute and relative reductions. Between 2020 and 2024, estimates indicate that stunting prevalence among children under two years declined in all districts, with absolute reductions ranging from five percentage points in Nyabihu and Nyaruguru to 27 percentage points in Kamonyi. The largest relative reductions were observed in Kamonyi (77.1%), Nyarugenge (73.9%), and Ngoma (71.4%), whereas the smallest occurred in Rubavu (14.6%) and Nyabihu (13.9%). By 2024, most districts had achieved prevalence levels below the national target of 19%, although elevated prevalence persisted in a few districts, including Rubavu (35%), Nyabihu (31%), Nyaruguru (28%), Burera (28%), and Ngororero (27%).

**Figure 3:**
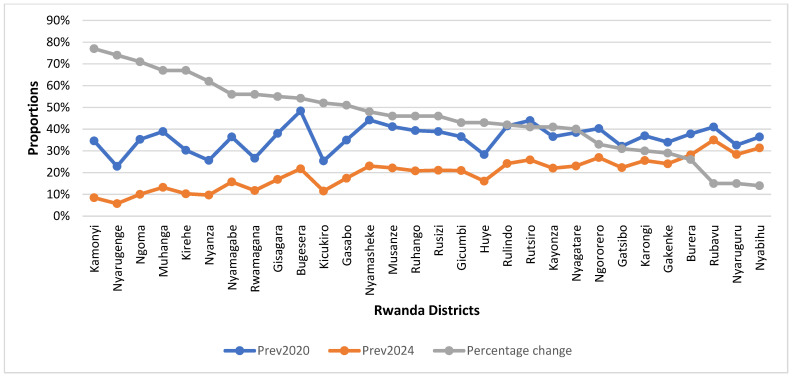
Trends and changes in the prevalence of stunting among the population of children under two years old per district in Rwanda between 2020 and 2024.

However, the reduction in the prevalence of stunting among the population of children under two years old in Rwanda between 2020 and 2024 was heterogeneously distributed across the country, with some districts and sectors making substantial reductions, while other administrative areas struggled to make progress in the prevention and control of stunting (Figure 3). The highest proportional reduction in the prevalence of stunting among the population of children under two years old in Rwanda was achieved in Kamonyi, Nyarugenge, and Ngoma districts, reporting a reduction of over 70% (Figure 2). A small decrease in stunting prevalence was achieved in districts such as Rubavu (15%), Nyaruguru (15%), and Nyabihu (14%), as is shown in Figure 3.

Our refined spatiotemporal analysis of the prevalence of stunting among children under two years old in Rwanda at the sector level revealed that the point and proportional change in stunting prevalence was heterogeneous across the different sectors of the country.

**Figure 4:**
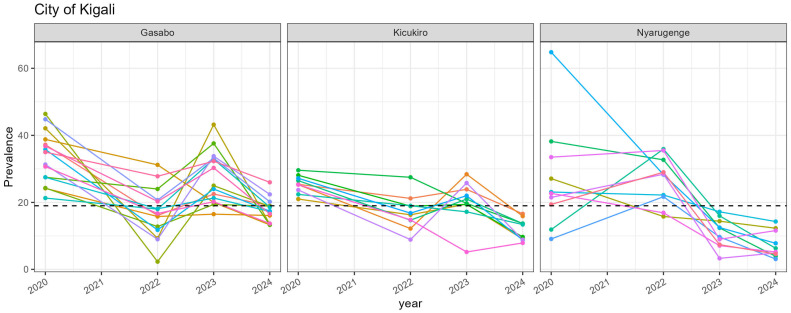
Trends in the prevalence of stunting among children under two years old in the sectors of the central province of Rwanda between 2020 and 2024. Different colors represent the sectors within a given district.

Color variation was used to present prevalence patterns at a sector level within a given district. The central province, also known as Kigali City, capital of Rwanda, is made up of three districts and 35 sectors; Kicukiro district contains 10 sectors; Gasabo district has 15 sectors; Nyarugenge contains 10 sectors. Figure 4 shows the stunting prevalence trend per sector. While the sectors in Nyarugenge district are mainly characterized by a steady decrease in stunting prevalence among children under two years old, the prevalence of stunting fluctuated in the sectors in the other two districts (Gasabo and Kicukiro (Figure 4)). It is important to highlight that all sectors in Kicukiro and Nyarugenge had reduced the prevalence of stunting among the population of children under two years old to below the national target for 2024 (19%).

Figure S1, attached in the Supplementary Materials, shows the trends in the prevalence of stunting among children under two years old in the sectors of the Northern province of Rwanda between 2020 and 2024. A lower reduction was achieved in the prevalence of stunting among the population of children under two years old between 2020 and 2024 in the Northern province, with the highest reduction occurring in sectors belonging to Musanze, Gicumbi, and Rulindo districts, with 46%, 43%, and 42% reductions, respectively. Excluding the sectors in Burera, all sectors in the Northern province achieved a steady reduction in stunting prevalence between 2020 and 2024. Apparently, most of the sectors in the Northern province are still reporting a higher prevalence of stunting than the national target among the population of children under two years old.

**Figure 5:**
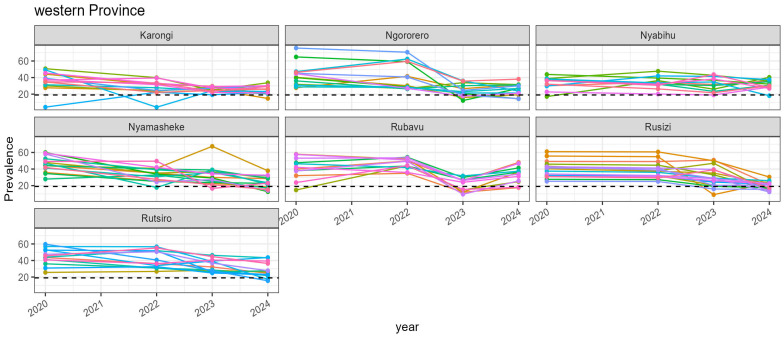
Trends of the prevalence of stunting among children under two years old in the sectors of the Western province of Rwanda between 2020 and 2024. Different colors represent the sectors within a given district.

The least progress in reducing the prevalence of stunting among children under two years old in Rwanda between 2020 and 2024 was reported by the sectors in the Western province, where sectors in Rubavu and Nyabihu districts reported the lowest proportional changes in stunting prevalence, at 15% and 14%, respectively (Figure 5). Overall, most sectors in this province did not achieve the national target of reducing the prevalence of stunting among children under two years old to below 19%. In contrast, most of the sectors in Rubavu, Nyabihu, and Ngororero districts reported an increase in the prevalence between 2023 and 2024.

Figure S2 in the Supplementary Materials presents the trend in the prevalence of stunting among children under two years old in the sectors of the Eastern province of Rwanda between 2020 and 2024. In the Eastern province, most of the reduction was achieved in Ngoma district, with 71%, followed by 67% and 56% reductions in stunting prevalence in Kirehe and Rwamagana, respectively, between 2020 and 2024 (Figure S2). Out of the seven districts in the Eastern province, most of the sectors in Kirehe, Ngoma, and Rwamagana districts achieved the national target for reducing the prevalence of stunting among the population of children under two years old to below 19% by 2024.

Figure S3 in the Supplementary Materials presents the Southern province, with a high fluctuation in the prevalence of stunting among children under two years old. A substantial reduction in prevalence was achieved across most of the sectors, with Kamonyi, Muhanga, and Nyanza districts reporting 77%, 67%, and 62% reductions in prevalence, respectively, overall (Figure S3). While sectors belonging to Nyaruguru district reported an increase in prevalence in the period between 2023 and 2024, during the same time period, sectors belonging to Nyanza met the national target by reducing the prevalence of stunting among children under two years old to below 19% in 2024.

## 4. Discussion

The spatiotemporal analysis of stunting prevalence among children under two years old in Rwanda represents a significant contribution to our understanding of the dynamics of childhood malnutrition within the country. This analysis is the first of its kind to aggregate and analyze the most recent data at the district and sector levels, providing valuable insights into the scale of stunting and its fluctuation over time.

The study observed a general decline in stunting prevalence across most districts and sectors between 2020 and 2024, consistent with the national objectives aimed at reducing malnutrition and improving child health outcomes through targeted interventions to address the burden of stunting in children under two years old in Rwanda. The study attributes the decline in the prevalence of stunting in Rwanda to various factors, including maternal and health services, socioeconomic improvements, nutrition programs, environmental and sanitation conditions, continuous screening, breastfeeding, education of mothers, access to healthcare services, access to water, hygiene, and sanitation, a decline in gender-based violence, access to family planning, antenatal care services, and a decline in teenage pregnancies [9]. In addition, there are also community programs, including community health insurance uptake, community health worker services, and early childhood development centers (ECDs), and social and economic factors aimed at reducing poverty and increasing food security. These interventions include the provision of shelters and free cows to poor families (also known as Girinka), house kitchen–garden programs (also known as Akarima k’igikoni), and the provision of food supplementation [11,12,14].

Despite overall progress, notable disparities persist, with certain districts and sectors continuing to exhibit alarmingly high stunting prevalence that requires immediate and targeted interventions. While the three districts in Kigali (Nyarugenge, Kicukiro, and Gasabo) have recorded substantial improvements, sectors in the Western provinces continue to show high stunting rates. Several factors likely contribute to these persistent patterns. Geographic and environmental challenges limit households to subsistence farming, and seasonal food shortages are common due to the limited arable land. Water, sanitation, and hygiene (WASH) issues are also prevalent; in the Western province, only 35% of households used appropriate water treatment methods, and access to clean water and adequate toilet facilities is limited. Land degradation due to soil erosion and poor road access in districts such as Nyamasheke, Rutsiro, and Rusizi limit production and population movements, including access to healthcare services. Additionally, climate-change-induced landslides have a severe effect on food diversity and security in the region [21,22]. Furthermore, inadequate nutrient intake in food for both mothers and children is common in the Western province. Other reasons include persistently high poverty rates and family sizes, as well as poor water, sanitation, and hygiene, leading to a high burden of diarrheal diseases, intestinal worms, and nutrient loss, contributing to stunting [4,21,22,23].

Moreover, well-established evidence shows a critical connection between the under-two-year period of life and long-term development outcomes, underscoring the urgency of prioritized interventions in maternal and child health [24]. The WHO classification of stunting provides a clear standard for identifying affected children, yet the response requires more than identification; it must include comprehensive support systems that encompass food security, healthcare access, and education on optimal nutritional practices [24,25].

Interestingly, in the NST2 [25] and HSSPV [11], the Government of Rwanda demonstrated Rwanda’s commitment to decreasing the proportion of children affected by stunting from 33% to 15% by 2029. To meet these ambitious targets, the interventions must be responsive and informed by evidence on the unique challenges faced by different sectors. The data suggest that the successful implementation of community-based targeted interventions at the local level, focusing on specific risk factors such as inadequate food intake, recurrent illnesses, including infectious diseases, household income, and poor maternal health, negatively affect stunting reduction efforts [26,27].

Furthermore, as this study used the dataset from the national continuous nutrition screening carried out by community health workers, it reaffirms the necessity of continuous screening, the significant role of CHWs in ongoing efforts, and the importance of utilizing consistent, high-quality data collection methods to update nutritional status. Multi-sectoral collaboration, including efforts from government agencies, NGOs, and community-based organizations, is essential for fostering an integrated response to child malnutrition.

While this study benefits from the use of up-to-date routine surveillance data encompassing all districts and sectors in Rwanda, several limitations should be noted. First, data for 2021 were unavailable due to a temporary suspension of surveillance during the COVID-19 pandemic, which may have affected the temporal trend analysis. Second, the dataset contained a limited number of variables and did not capture key indicators such as food security, dietary diversity, illness history, or uptake of implemented nutritional interventions; therefore, the results should not be interpreted as causal. Third, children were not consistently followed as individuals across multiple screenings, which limited the ability to assess longitudinal changes or the persistence of stunting under two years of age at the individual level. These limitations were taken into account when interpreting the results and underscore the need for a more comprehensive, longitudinally linked stunting surveillance system [14,28].

## 5. Conclusions

In summary, while this study demonstrates progress in reducing stunting in Rwanda, it also highlights persistent challenges. Addressing stunting requires a coordinated, evidence-based approach that accounts for the temporal and spatial variations in malnutrition across the country. Future research should prioritize continuous screening and evaluation to monitor progress, identify sector-level determinants, and address emerging challenges related to child nutrition. Further investigation is needed into the role of infectious disease burden on stunting, the impact of socio-cultural beliefs and practices on child growth, and the perspectives of communities and stakeholders. By fostering a collaborative environment and implementing sector-specific, evidence-based interventions, Rwanda can make substantial strides in reducing stunting and creating a healthier, more productive population.

## Figures and Tables

**Table 1:** The WHO prevalence thresholds for stunting in children.

Indicator	Prevalence Cut-Off Values for Public Health Significance
Stunting	<10% = low
	10 to <19% = moderate
	20 to <29% = high
	≥30% = very high

**Table 2:** Samples per district and per year.

		Year			
	Population per District	2020	2022	2023	2024
1	Bugesera	11,612	8898	20,039	17,763
2	Burera	11,470	8198	11,988	10,915
3	Gakenke	14,354	9296	12,661	12,309
4	Gasabo	3899	10,949	10,054	14,566
5	Gatsibo	20,277	9026	14,091	12,575
6	Gicumbi	15,702	12,954	14,072	14,848
7	Gisagara	12,186	10,167	14,368	13,863
8	Huye	10,452	10,534	12,001	12,578
9	Kamonyi	9737	7664	11,988	13,156
10	Karongi	13,268	9381	10,949	10,598
11	Kayonza	11,518	10,482	10,248	11,349
12	Kicukiro	8751	4713	8125	9744
13	Kirehe	13,079	10,956	15,617	15,973
14	Muhanga	9083	5058	9824	10,710
15	Musanze	16,336	8719	12,995	14,298
16	Ngoma	12,668	12,681	14,341	15,945
17	Ngororero	12,696	7779	10,652	11,326
18	Nyabihu	9615	8290	9181	11,352
19	Nyagatare	22,351	22,371	18,237	17,858
20	Nyamagabe	11,595	10,216	11,503	12,935
21	Nyamasheke	16,415	9716	11,571	11,611
22	Nyanza	8176	5803	11,393	6307
23	Nyarugenge	8280	5675	7278	9729
24	Nyaruguru	13,003	8840	11,289	12,131
25	Rubavu	17,067	16,255	15,167	15,014
26	Ruhango	13,088	13,121	10,845	10,784
27	Rulindo	11,223	6527	8746	10,690
28	Rusizi	11,952	11,943	13,749	15,412
29	Rutsiro	13,791	7924	10,603	11,683
30	Rwamagana	13,717	13,720	15,154	16,680
TOTAL		379,381	299,878	370,752	386,726

**Table 3:** Stunting prevalence (%) among children under two years of age by district and year, along with corresponding absolute and relative reductions between 2020 and 2024.

District	Stunting Prevalence 2020	Stunting Prevalence 2022	Stunting Prevalence 2023	Stunting Prevalence 2024	Absolute Reduction	Relative Reduction
Bugesera	48	36	30	22	26	54.2
Burera	38	34	30	28	10	26.3
Gakenke	34	32	28	24	10	29.4
Gasabo	35	17	25	17	18	51.4
Gatsibo	32	26	25	22	10	31.2
Gicumbi	37	33	29	21	16	43.2
Gisagara	38	36	23	17	21	55.3
Huye	28	40	16	16	12	42.9
Kamonyi	35	25	23	8	27	77.1
Karongi	37	28	25	26	11	29.7
Kayonza	37	27	31	22	15	40.5
Kicukiro	25	17	20	12	13	52.0
Kirehe	30	30	15	10	20	66.7
Muhanga	39	30	17	13	26	66.7
Musanze	41	33	26	22	19	46.3
Ngoma	35	35	30	10	25	71.4
Ngororero	40	30	23	27	13	32.5
Nyabihu	36	33	33	31	5	13.9
Nyagatare	38	37	33	23	15	39.5
Nyamagabe	36	33	28	16	20	55.6
Nyamasheke	44	34	30	23	21	47.7
Nyanza	26	24	26	10	16	61.5
Nyarugenge	23	28	11	6	17	73.9
Nyaruguru	33	34	23	28	5	15.2
Rubavu	41	47	25	35	6	14.6
Ruhango	39	39	27	21	18	46.2
Rulindo	41	35	32	24	17	41.5
Rusizi	39	37	29	21	18	46.2
Rutsiro	44	36	32	26	18	40.9
Rwamagana	27	26	19	12	15	55.6

## Data Availability

All the data used in this study are available from the Rwanda National Public Health Institute, also known as the Rwanda Biomedical Center (RBC), upon reasonable request. Available online: https://rbc.gov.rw (accessed on 2 December 2024).

## Supplementary Materials

The following supporting information can be downloaded at: https://www.mdpi.com/article/10.3390/nu17172808/s1, Figure S1. Trends in the prevalence of stunting among children under two years old in the sectors of the Northern province of Rwanda between 2020 and 2024; Figure S2. Trends in the prevalence of stunting among children under two years old in the sectors of the Eastern province of Rwanda between 2020 and 2024; Figure S3. Trends in the prevalence of stunting among children under two years old in the sectors of the Southern province of Rwanda between 2020 and 2024; Table S1. Model-based estimates of stunting prevalence among children under two years old for each sector in Rwanda, with the lower and upper bounds of the 95% credible intervals for the years 2020, 2022, 2023, and 2024.

## Abbreviations

The following abbreviations are used in this manuscript:

BYM	Besag–York–Mollié
CHW	Community Health Works
CPO	Conditional Predictive Ordinate
DHMT	District Health Management Team
DIC	Deviance Information Criterion
ECD	Early Childhood Development
HAZ	Height-for-Age Z-score
HSSP	Health Sector Strategic Plan
ICAR	Intrinsic conditional autoregressive
INLA	Integrated Nested Laplace Approximation
LMIC	Low- and Middle-Income Countries
MCCH	Maternal Child Health and Community Health
MINECOFIN	Ministry of Finance and Economic Planning
MoH	Ministry of Health
MUAC	Middle Upper Arm Circumference
NCD	Non-Communicable Disease
NGO	Non-Governmental Organization
NMCH	National Maternal and Child Health
NST	National Strategy for Transformation
RBC	Rwanda Biomedical Center
RDHS	Rwanda Demographic and Health Survey
RW1	First-order random walk
SD	Standard Deviation
WAIC	Watanabe–Akaike Information Criterion
WHO	World Health Organization
