# Supplementary material for: Spatiotemporal Trends in Stunting Prevalence Among Children Aged Two Years Old in Rwanda (2020–2024): A Retrospective Analysis
Source: Nutrients. 2025 Aug 29;17(17):2808. doi: 10.3390/nu17172808 (PMC12430221; doi:10.3390/nu17172808)
Supplement: Supplementary file 1 [file nutrients-17-02808-s001.zip › nutrients-3790349-supplementary.pdf]

Supplementary Materials

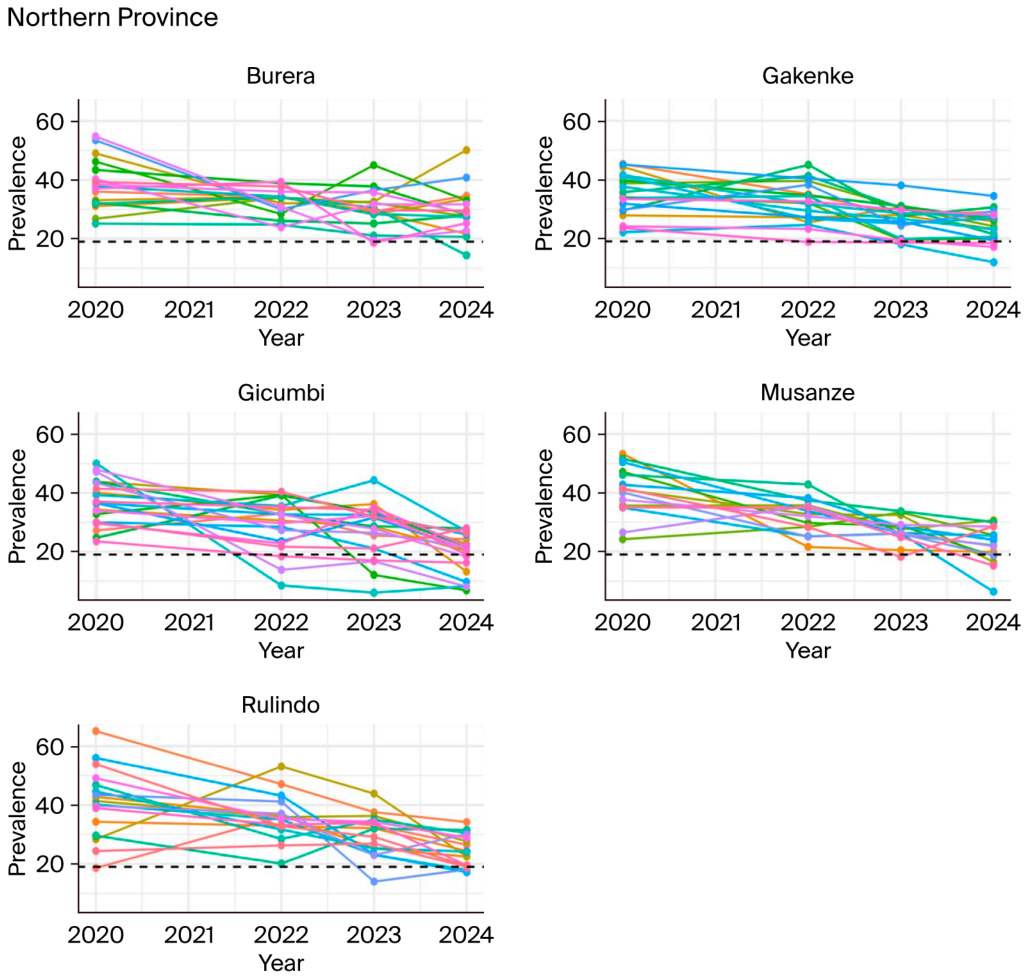

Figure S1. Trends in the prevalence of stunting among children under two years old in the sectors of the Northern province of Rwanda between 2020 and 2024.

A lower reduction in the prevalence of stunting among the population of children under two years old between 2020 and 2024 was achieved in the Northern province, with the highest reductions occurring in sectors belonging to Musanze, Gicumbi, and Rulindo districts, with relative reductions of 46%, 43%, and 42%, respectively (Figure S2). Excluding Burera sector, all sectors in the Northern province achieved a steady reduction in stunting prevalence between 2020 and 2024 (Figure S1). Apparently, most of the sectors in the Northern province are still reporting a prevalence of stunting among the population of children under two years old that is higher than the national target.

Eastern Province

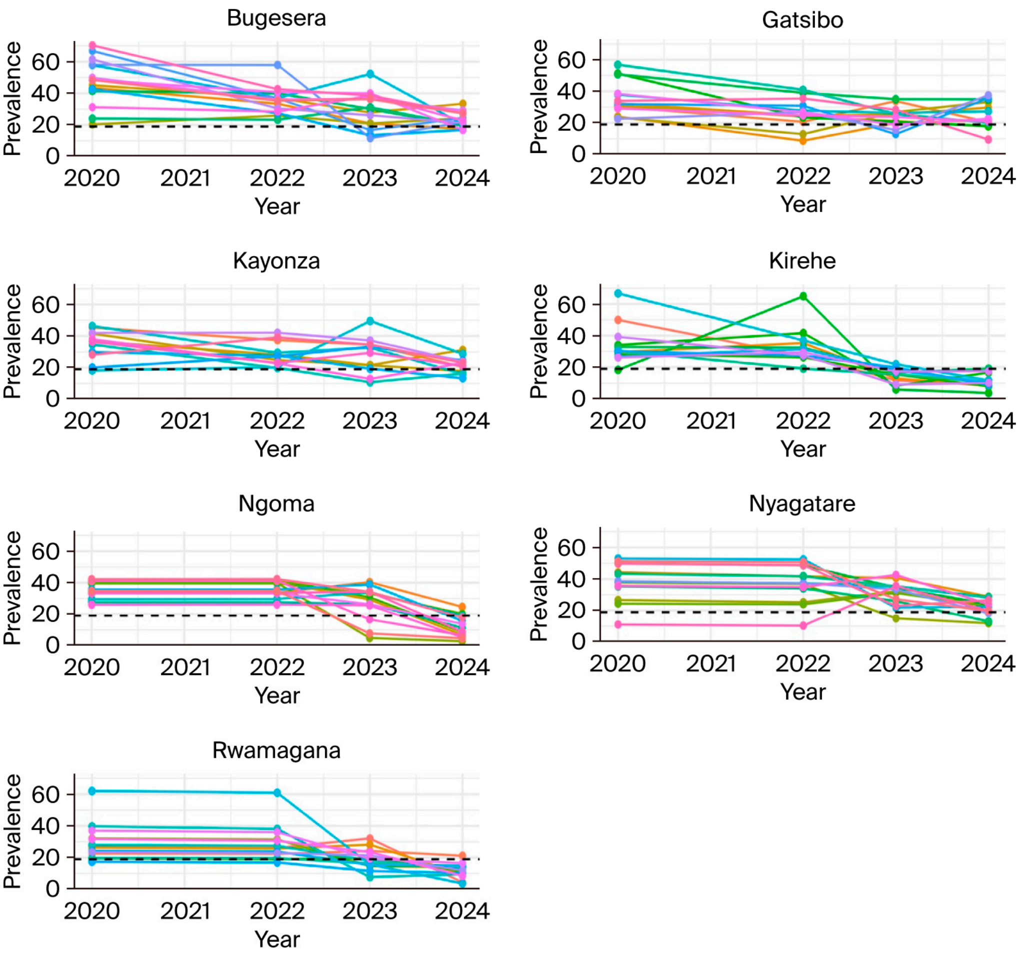

Figure S2 Trends in the prevalence of stunting among children under two years old in the sectors of the Eastern province of Rwanda between 2020 and 2024.

In the Eastern province, most of the reduction was achieved in Ngoma district, with 71%, followed by 67% and 56% reductions in stunting prevalence in Kirehe and Rwamagana, respectively, between 2020 and 2024 (Figure S1). Out of the seven districts in the Eastern province, most of the sectors in Kirehe, Ngoma, and Rwamagana districts achieved the national target for reducing the prevalence of stunting among the population of children under two years old to below 19% by 2024.

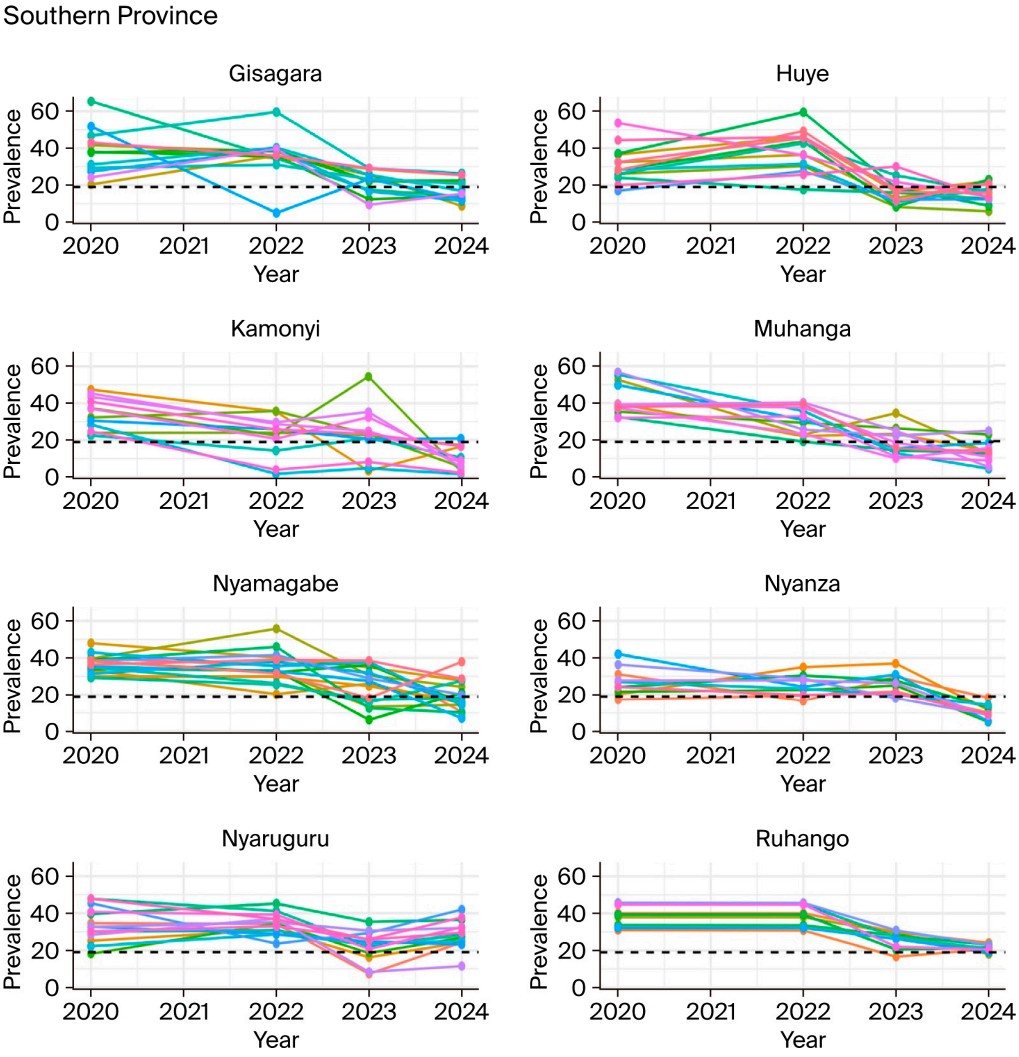

Figure S3. Trends in the prevalence of stunting among children under two years old in the sectors of the Southern province of Rwanda between 2020 and 2024.

A notable fluctuation in the prevalence of stunting among children under two years old occurred in the sectors of the Southern province. A substantial reduction in prevalence was achieved across most of the sectors, with Kamonyi, Muhanga, and Nyanza districts reporting relative reductions of 77%, 67%, and 62% in prevalence, respectively, overall (Figure S3). Sectors belonging to Nyaruguru district reported an increase in prevalence in the period between 2023 and 2024. During the same time period, sectors belonging to Nyanza met the national target by reducing the prevalence of stunting among children under two years old to below 19% in 2024.

Table S1. Model-based estimates of stunting prevalence among children under two years old for each sector in Rwanda, with the lower and upper bounds of the 95% credible intervals for the years 2020, 2022, 2023, and 2024.

No.	District	Sector	Prev2020_Low	Prev 2020	Prev2020_Upper	Prev2022_Low	Prev 2022	Prev2022_Upper	Prev2023_Low	Prev 2023	Prev2023_Upper	Prev2024_Low	Prev 2024	Prev2024_Upper
1		Gashora	0.1873	0.2089	0.2323	0.1529	0.1744	0.1981	0.4033	0.434	0.4652	0.2964	0.3342	0.3744
2		Juru	0.2548	0.2782	0.3029	0.3072	0.3337	0.3612	0.4225	0.4512	0.4801	0.3303	0.3703	0.4121
3		Kamabuye	0.1849	0.209	0.2353	0.2152	0.2419	0.2708	0.1754	0.2034	0.2346	0.228	0.259	0.2926
4		Mareba	0.2743	0.3002	0.3275	0.1709	0.1949	0.2213	0.3813	0.4141	0.4477	0.3607	0.3987	0.438
5		Mayange	0.2926	0.3114	0.3309	0.18	0.1976	0.2165	0.2196	0.24	0.2617	0.2027	0.2334	0.2671
6	Bugesera	Musenyi	0.4963	0.5217	0.5471	0.1807	0.2032	0.2276	0.541	0.5778	0.6137	0.3369	0.3702	0.4048
7		Mwogo	0.1154	0.1336	0.1543	0.147	0.1678	0.1908	0.3864	0.4261	0.4668	0.2424	0.2745	0.309
8		Ngeruka	0.148	0.1668	0.1873	0.2209	0.2439	0.2685	0.6429	0.6681	0.6924	0.3367	0.368	0.4005
9		Ntarama	0.101	0.1159	0.1326	0.1966	0.2177	0.2403	0.5378	0.5802	0.6214	0.5363	0.5787	0.6201
10		Nyamata	0.2444	0.2614	0.2792	0.1953	0.2133	0.2326	0.5647	0.6136	0.6603	0.1527	0.3038	0.5135
11		Nyarugenge	0.3354	0.3654	0.3964	0.264	0.2915	0.3206	0.4274	0.4979	0.5685	0.3245	0.3602	0.3975

12		Rilima	0.3723	0.3998	0.428	0.1478	0.1675	0.1894	0.4514	0.4836	0.5158	0.3667	0.4081	0.4508
13		Ruhuha	0.3567	0.3849	0.4139	0.2059	0.2284	0.2526	0.286	0.3118	0.3388	0.255	0.2844	0.3158
14		Rweru	0.3601	0.3852	0.411	0.2511	0.2744	0.299	0.6567	0.703	0.7454	0.3901	0.426	0.4626
15		Shyara	0.3268	0.3632	0.4014	0.2393	0.2732	0.3099	0.4257	0.4843	0.5434	0.3094	0.3532	0.3996
16		Bungwe	0.2595	0.2924	0.3276	0.3032	0.346	0.3914	0.3196	0.3602	0.403	0.3029	0.3424	0.3842
17		Butaro	0.2689	0.292	0.3162	0.188	0.2169	0.2488	0.2768	0.3113	0.3481	0.2949	0.3405	0.3893
18		Cyanika	0.2598	0.2827	0.3069	0.3081	0.3347	0.3624	0.3052	0.3311	0.3581	0.3156	0.342	0.3693
19		Cyeru	0.2828	0.3264	0.3732	0.4561	0.5013	0.5466	0.4428	0.4902	0.5378	0.2743	0.3198	0.369
20		Gahunga	0.2883	0.3217	0.3569	0.2441	0.2765	0.3114	0.2429	0.2681	0.295	0.3058	0.3403	0.3766
21		Gatebe	0.4134	0.4503	0.4878	0.2956	0.3299	0.366	0.4269	0.4622	0.4978	0.2473	0.2842	0.3243
22		Gitovu	0.3319	0.3785	0.4274	0.2391	0.2815	0.3281	0.3911	0.4339	0.4778	0.3326	0.3894	0.4493
23		Kagogo	0.22	0.2517	0.2864	0.2465	0.2793	0.3146	0.2908	0.3226	0.3561	0.2145	0.2612	0.3139
24	Burera	Kinoni	0.1764	0.2109	0.2501	0.1731	0.2066	0.2446	0.2178	0.2516	0.2887	0.2022	0.2476	0.2992
25		Kinyababa	0.2749	0.3063	0.3395	0.1223	0.1438	0.1685	0.2887	0.3187	0.3504	0.3033	0.3406	0.38
26		Kivuye	0.2504	0.2836	0.3194	0.2412	0.2738	0.309	0.339	0.3781	0.4189	0.305	0.343	0.3831
27		Nemba	0.3288	0.3651	0.403	0.372	0.4084	0.446	0.4929	0.5348	0.5762	0.2599	0.2999	0.3432
28		Rugarama	0.1687	0.1948	0.2237	0.2006	0.2278	0.2575	0.5106	0.5479	0.5846	0.2701	0.308	0.3487
29		Rugengabari	0.2813	0.3171	0.3552	0.2753	0.3167	0.3612	0.3543	0.403	0.4536	0.1924	0.2386	0.2918
30		Ruhunde	0.3214	0.3572	0.3946	0.2568	0.2861	0.3173	0.3515	0.3849	0.4193	0.3193	0.3601	0.4031
31		Rusarabuye	0.1602	0.1859	0.2147	0.2173	0.2529	0.2921	0.3362	0.3708	0.4069	0.3557	0.3945	0.4346
32		Rwerere	0.2582	0.2956	0.3359	0.2621	0.296	0.3323	0.3567	0.3935	0.4315	0.3324	0.3781	0.4261
33		Busengo	0.2482	0.2795	0.3131	0.2582	0.29	0.3241	0.4231	0.4532	0.4835	0.3153	0.3501	0.3865
34		Coko	0.2469	0.2791	0.3137	0.1982	0.2306	0.2666	0.2468	0.278	0.3117	0.2255	0.2713	0.3224
35		Cyabingo	0.2718	0.3068	0.3442	0.2255	0.2569	0.2909	0.4083	0.4426	0.4774	0.2201	0.2538	0.2908
36		Gakenke	0.277	0.3055	0.3355	0.2135	0.2406	0.27	0.3573	0.3869	0.4173	0.3595	0.3951	0.4318
37		Gashenyi	0.1691	0.1947	0.2233	0.1739	0.2005	0.2299	0.3114	0.3396	0.369	0.2795	0.3203	0.364
38		Janja	0.2721	0.3099	0.3504	0.231	0.2653	0.3028	0.3603	0.3968	0.4345	0.2923	0.346	0.404
39		Kamubuga	0.2452	0.275	0.3069	0.2746	0.3055	0.3384	0.2636	0.2954	0.3292	0.4186	0.4502	0.4822
40		Karambo	0.2669	0.304	0.3439	0.1788	0.2141	0.2541	0.3182	0.3565	0.3967	0.3601	0.4118	0.4656
41		Kivuruga	0.1705	0.1976	0.2278	0.1774	0.205	0.2356	0.3042	0.3372	0.3719	0.3067	0.3441	0.3834
42	Gakenke	Mataba	0.2516	0.2859	0.323	0.2405	0.2755	0.3136	0.3766	0.415	0.4545	0.276	0.3168	0.3605
43		Minazi	0.23	0.2658	0.305	0.1967	0.2313	0.2699	0.3351	0.3749	0.4164	0.2466	0.2941	0.3465
44		Mugunga	0.2177	0.2495	0.2842	0.2363	0.2694	0.3051	0.3778	0.4106	0.4443	0.221	0.2674	0.3195
45		Muhondo	0.1527	0.18	0.2111	0.0992	0.1192	0.1425	0.1941	0.2209	0.2501	0.214	0.2459	0.281
46		Muyongwe	0.2233	0.2567	0.2931	0.1615	0.1929	0.2288	0.282	0.3185	0.3573	0.2347	0.2718	0.3124
47		Muzo	0.3468	0.3802	0.4148	0.3096	0.3444	0.381	0.4196	0.4515	0.4837	0.3638	0.4031	0.4435
48		Nemba	0.2105	0.2427	0.278	0.2585	0.2926	0.3293	0.2652	0.2968	0.3305	0.34	0.3821	0.4261
49		Ruli	0.1661	0.1932	0.2235	0.1466	0.1706	0.1978	0.2136	0.2412	0.2712	0.2	0.2324	0.2683
50		Rusasa	0.2646	0.2967	0.3308	0.2454	0.2793	0.316	0.3008	0.3337	0.3682	0.2836	0.3263	0.3722
51		Rushashi	0.1578	0.1857	0.2173	0.1553	0.1844	0.2176	0.2035	0.2351	0.2698	0.1532	0.1878	0.228
52		Bumbogo	0.2057	0.2247	0.2449	0.1752	0.1895	0.2047	0.2969	0.3717	0.4532	0.1421	0.1611	0.1822
53		Gatsata	0.142	0.1652	0.1913	0.1395	0.1612	0.1855	0.2112	0.2421	0.2759	0.1329	0.1584	0.1878
54		Gikomero	0.1617	0.1998	0.2444	0.1046	0.1329	0.1674	0.2226	0.3883	0.5848	0.2574	0.3119	0.3721
55		Gisozi	0.3902	0.4319	0.4745	0.1101	0.1347	0.1637	0.2955	0.4211	0.5579	0.0739	0.093	0.1163
56		Jabana	0.2268	0.2504	0.2755	0.17	0.1885	0.2084	0.4123	0.4643	0.5171	0.0165	0.0229	0.0317
57		Jali	0.1628	0.1943	0.2302	0.168	0.1894	0.2128	0.2058	0.243	0.2845	0.0963	0.1279	0.1678
58		Kacyiru	0.3197	0.3756	0.4351	0.098	0.1334	0.179	0.238	0.2749	0.3152	0.2054	0.2403	0.2791
59	Gasabo	Kimihurura	0.2697	0.3292	0.3947	0.1348	0.1863	0.2518	0.1787	0.2132	0.2525	0.148	0.1799	0.217
60		Kimironko	0.1574	0.2128	0.2811	0.1225	0.173	0.2388	0.1225	0.2754	0.5086	0.1313	0.1815	0.2455
61		Kinyinya	0.2201	0.2405	0.262	0.1587	0.1743	0.1911	0.2758	0.3575	0.4486	0.1027	0.1182	0.1355
62		Ndera	0.3084	0.3317	0.3559	0.1844	0.2024	0.2217	0.4079	0.4484	0.4896	0.1914	0.2073	0.2241
63		Nduba	0.2934	0.3384	0.3865	0.2043	0.224	0.245	0.208	0.3128	0.4409	0.0671	0.0904	0.1207
64		Remera	0.1575	0.2018	0.2547	0.0979	0.1375	0.1897	0.1416	0.3069	0.5435	0.1269	0.1673	0.2174
65		Rusororo	0.2595	0.3032	0.3507	0.1396	0.1662	0.1969	0.2265	0.368	0.5366	0.1752	0.2027	0.2332
66		Rutunga	0.2699	0.3226	0.3801	0.2209	0.2603	0.304	0.3141	0.3497	0.3871	0.2191	0.2783	0.3464
67		Gasange	0.2966	0.3376	0.3813	0.171	0.2054	0.2447	0.2703	0.3021	0.3359	0.1631	0.2067	0.2583
68		Gatsibo	0.1761	0.1985	0.2231	0.196	0.2184	0.2426	0.2154	0.24	0.2664	0.0632	0.087	0.1185
69		Gitoki	0.2326	0.2534	0.2753	0.2699	0.2973	0.3261	0.3018	0.3239	0.3469	0.2069	0.2456	0.2889
70		Kabarore	0.1934	0.2121	0.2322	0.1566	0.178	0.2015	0.2927	0.3094	0.3266	0.2168	0.2409	0.2668
71		Kageyo	0.2368	0.269	0.3038	0.2938	0.3322	0.3728	0.2108	0.2352	0.2614	0.0993	0.1294	0.167
72		Kiramuruzi	0.1819	0.2061	0.2326	0.1589	0.1798	0.2028	0.4853	0.5123	0.5393	0.2068	0.2349	0.2656
73	Gatsibo	Kiziguro	0.3217	0.3499	0.3792	0.3211	0.3487	0.3774	0.4793	0.5065	0.5337	0.3557	0.3896	0.4246
74		Muhura	0.233	0.2562	0.2809	0.1748	0.2006	0.2292	0.5443	0.5684	0.5922	0.3679	0.4085	0.4504
75		Murambi	0.2338	0.2622	0.2927	0.2433	0.2727	0.3042	0.357	0.3794	0.4023	0.2487	0.2784	0.3102
76		Ngarama	0.1068	0.1287	0.1544	0.3259	0.3564	0.3881	0.2969	0.319	0.342	0.287	0.3089	0.3316
77		Nyagihanga	0.1331	0.1549	0.1794	0.3453	0.3754	0.4065	0.2015	0.2265	0.2536	0.2232	0.2669	0.3156
78		Remera	0.1644	0.1883	0.2147	0.198	0.2272	0.2594	0.3585	0.3841	0.4104	0.1916	0.268	0.3611
79		Rugarama	0.2172	0.2383	0.2608	0.1855	0.2094	0.2354	0.2686	0.2909	0.3143	0.2219	0.2484	0.277
80		Rwimbogo	0.2475	0.2829	0.3213	0.0785	0.0954	0.1154	0.3158	0.3387	0.3625	0.3198	0.3537	0.3891
81	Gicumbi	Bukure	0.222	0.2547	0.2905	0.2085	0.2417	0.2782	0.2446	0.2721	0.3015	0.2961	0.3303	0.3664

82		Bwisige	0.3248	0.362	0.401	0.1086	0.1317	0.1588	0.3628	0.4013	0.4411	0.3044	0.3421	0.3819
83		Byumba	0.262	0.2854	0.31	0.1825	0.2029	0.2249	0.3195	0.3431	0.3675	0.2786	0.3064	0.3356
84		Cyumba	0.2964	0.3339	0.3737	0.1954	0.2265	0.2608	0.3985	0.4384	0.479	0.3224	0.3944	0.4713
85		Giti	0.0988	0.1213	0.1481	0.0515	0.0679	0.0891	0.2934	0.328	0.3646	0.3446	0.3933	0.4442
86		Kageyo	0.2931	0.3344	0.3784	0.1913	0.2179	0.2471	0.2209	0.2466	0.2743	0.3358	0.3914	0.45
87		Kaniga	0.2522	0.286	0.3223	0.245	0.2789	0.3155	0.4033	0.4384	0.4742	0.2975	0.3285	0.361
88		Manyagiro	0.0465	0.0605	0.0783	0.0651	0.0818	0.1023	0.4655	0.5	0.5344	0.0701	0.0854	0.1038
89		Miyove	0.4065	0.4433	0.4807	0.2386	0.269	0.3018	0.3597	0.3934	0.4282	0.3088	0.3551	0.4044
90		Mukarange	0.2904	0.3264	0.3646	0.2267	0.2576	0.2912	0.327	0.3645	0.4038	0.2944	0.3267	0.3608
91		Muko	0.1812	0.2107	0.2436	0.0783	0.0971	0.1198	0.2628	0.2999	0.3399	0.2516	0.284	0.3188
92		Mutete	0.2827	0.3165	0.3524	0.1838	0.2091	0.2368	0.3359	0.3653	0.3958	0.1959	0.2352	0.2796
93		Nyamiyaga	0.2282	0.2638	0.3027	0.1861	0.2163	0.2499	0.3922	0.4336	0.476	0.2314	0.2726	0.3182
94		Nyankenke	0.1438	0.1666	0.1923	0.0635	0.0809	0.1025	0.4378	0.4719	0.5062	0.1145	0.1377	0.1648
95		Rubaya	0.237	0.2778	0.3226	0.1486	0.1832	0.2236	0.4304	0.4795	0.529	0.282	0.326	0.3732
96		Rukomo	0.2934	0.3223	0.3526	0.1839	0.2096	0.238	0.3113	0.3396	0.3692	0.272	0.2973	0.3239
97		Rushaki	0.3113	0.3504	0.3916	0.1839	0.2189	0.2585	0.2623	0.2961	0.3324	0.1965	0.2282	0.2634
98		Rutare	0.1472	0.1696	0.1947	0.1405	0.1621	0.1863	0.2112	0.2354	0.2616	0.1589	0.1837	0.2113
99		Ruvune	0.3081	0.3422	0.378	0.1623	0.1896	0.2204	0.3373	0.3705	0.405	0.3175	0.3517	0.3874
100		Rwamiko	0.1749	0.2107	0.2515	0.2415	0.2797	0.3214	0.2618	0.3003	0.3419	0.1874	0.2175	0.2511
101		Shangasha	0.2819	0.3185	0.3576	0.2304	0.2633	0.2991	0.3709	0.4144	0.4592	0.3661	0.4041	0.4434
102		Gikonko	0.2618	0.2878	0.3152	0.2303	0.2548	0.2811	0.3945	0.4309	0.4681	0.3107	0.3448	0.3806
103		Gishubi	0.2324	0.2561	0.2815	0.0725	0.0869	0.1037	0.1819	0.2038	0.2276	0.3335	0.3604	0.3882
104		Kansi	0.1954	0.2214	0.2497	0.1896	0.2163	0.2456	0.3853	0.4174	0.4502	0.338	0.3811	0.426
105		Kibirizi	0.107	0.1242	0.1437	0.1227	0.1413	0.1622	0.3517	0.3803	0.4099	0.3285	0.3566	0.3858
106		Kigembe	0.1969	0.2243	0.2544	0.1995	0.2255	0.2538	0.3462	0.3778	0.4104	0.3481	0.39	0.4335
107		Mamba	0.1562	0.1738	0.1928	0.123	0.1405	0.1599	0.6108	0.6541	0.6949	0.3184	0.3457	0.374
108	Gisagara	Muganza	0.2674	0.2918	0.3174	0.239	0.2636	0.2897	0.4411	0.468	0.4951	0.5551	0.5959	0.6354
109		Mugombwa	0.2114	0.233	0.256	0.1876	0.2091	0.2324	0.2683	0.2925	0.3179	0.2768	0.311	0.3473
110		Mukindo	0.2314	0.2572	0.2847	0.1487	0.1688	0.1912	0.2771	0.3119	0.349	0.375	0.4033	0.4323
111		Musha	0.1462	0.1669	0.1898	0.0989	0.1165	0.1367	0.2468	0.2747	0.3046	0.3621	0.3907	0.4201
112		Ndora	0.2107	0.2343	0.2597	0.1074	0.1247	0.1443	0.4856	0.518	0.5502	0.0358	0.0504	0.0706
113		Nyanza	0.0731	0.0942	0.1206	0.129	0.1537	0.1821	0.2181	0.2433	0.2704	0.3573	0.3973	0.4387
114		Save	0.2674	0.2924	0.3188	0.2271	0.2558	0.2869	0.4005	0.4298	0.4596	0.3268	0.3605	0.3956
115		Gishamvu	0.1303	0.1623	0.2004	0.1904	0.2219	0.257	0.3068	0.3661	0.4298	0.4284	0.4634	0.4986
116		Huye	0.1133	0.1321	0.1534	0.1846	0.2083	0.2342	0.2961	0.3245	0.3543	0.3297	0.3653	0.4023
117		Karama	0.0649	0.0822	0.1035	0.0437	0.0591	0.0796	0.2318	0.2623	0.2954	0.2713	0.3033	0.3373
118		Kigoma	0.0692	0.085	0.1038	0.2058	0.231	0.2583	0.2483	0.2812	0.3166	0.4053	0.4374	0.4701
119		Kinazi	0.1633	0.1852	0.2093	0.0734	0.0885	0.1064	0.3437	0.3705	0.3981	0.5577	0.5955	0.6322
120		Maraba	0.1393	0.1609	0.1851	0.1068	0.1256	0.1471	0.2125	0.241	0.2719	0.1522	0.1761	0.2028
121	Huye	Mbazi	0.2308	0.2535	0.2776	0.1438	0.1625	0.1831	0.2302	0.2614	0.2952	0.3895	0.4273	0.466
122		Mukura	0.0883	0.1084	0.1324	0.1558	0.179	0.2048	0.2524	0.2845	0.3189	0.281	0.3147	0.3505
123		Ngoma	0.0998	0.1213	0.1466	0.1025	0.1242	0.1497	0.1391	0.1696	0.2052	0.238	0.2762	0.318
124		Ruhashya	0.1922	0.218	0.2463	0.117	0.138	0.1621	0.5023	0.5366	0.5706	0.3307	0.3618	0.3942
125		Rusatira	0.2726	0.3015	0.332	0.1118	0.1319	0.1549	0.1778	0.2003	0.2248	0.2314	0.2569	0.2842
126		Rwaniro	0.1541	0.179	0.207	0.1765	0.2043	0.2352	0.4016	0.4437	0.4866	0.4264	0.4622	0.4983
127		Simbi	0.0946	0.1152	0.1396	0.1839	0.2096	0.2378	0.2919	0.3248	0.3596	0.4201	0.4559	0.4923
128		Tumba	0.1392	0.1636	0.1913	0.1379	0.1592	0.1831	0.2542	0.2815	0.3105	0.4608	0.4928	0.5248
129		Gacurabwenge	0.0232	0.0331	0.0469	0.1418	0.1653	0.1918	0.4287	0.4737	0.5191	0.324	0.3545	0.3862
130		Karama	0.1922	0.2267	0.2653	0.1463	0.1734	0.2043	0.1899	0.2393	0.2968	0.203	0.24	0.2814
131		Kayenzi	0.202	0.2332	0.2675	0.0408	0.0544	0.072	0.286	0.3225	0.3613	0.3197	0.357	0.3961
132		Kayumbu	0.498	0.5436	0.5885	0.02	0.0315	0.0491	0.329	0.3732	0.4197	0.1834	0.2251	0.273
133		Mugina	0.1893	0.2107	0.2339	0.0896	0.1053	0.1234	0.2012	0.2253	0.2514	0.1181	0.1432	0.1727
134	Kamonyi	Musambira	0.0376	0.0465	0.0574	0.0115	0.0164	0.0233	0.2604	0.284	0.3089	0.0112	0.0172	0.0262
135		Ngamba	0.1706	0.2052	0.2449	0.1776	0.208	0.2421	0.2653	0.3058	0.3496	0.2218	0.259	0.2999
136		Nyamiyaga	0.3259	0.3521	0.3793	0.0418	0.0544	0.0706	0.4035	0.4337	0.4644	0.265	0.2971	0.3314
137		Nyarubaka	0.2215	0.2489	0.2785	0.0644	0.0803	0.0997	0.4185	0.4526	0.4871	0.249	0.2871	0.3284
138		Rugarika	0.2965	0.3244	0.3536	0.0773	0.089	0.1022	0.3458	0.3695	0.3939	0.1757	0.2067	0.2417
139		Rukoma	0.0657	0.0812	0.0999	0.0161	0.0232	0.0334	0.2162	0.2472	0.2812	0.0288	0.0391	0.0529
140		Runda	0.2161	0.2331	0.2511	0.1522	0.1671	0.1831	0.3816	0.408	0.4349	0.2268	0.2578	0.2914
141		Bwishyura	0.2324	0.2593	0.2881	0.1276	0.148	0.1709	0.2522	0.2772	0.3037	0.2185	0.2456	0.2749
142		Gashari	0.2653	0.2962	0.3292	0.2232	0.2555	0.2907	0.4039	0.4407	0.4781	0.2808	0.3174	0.3565
143		Gishyita	0.2517	0.2853	0.3213	0.174	0.2053	0.2406	0.2668	0.3001	0.3356	0.2061	0.2374	0.2719
144		Gitesi	0.2167	0.2482	0.2826	0.306	0.337	0.3695	0.4782	0.507	0.5359	0.3522	0.3995	0.4487
145	Karongi	Mubuga	0.2113	0.2408	0.273	0.1712	0.203	0.239	0.2791	0.3127	0.3484	0.2363	0.277	0.3217
146		Murambi	0.2115	0.2424	0.2761	0.2008	0.2318	0.266	0.4564	0.4934	0.5306	0.0289	0.0421	0.0608
147		Murundi	0.1683	0.1923	0.2188	0.1973	0.224	0.2532	0.0327	0.043	0.0565	0.1964	0.2233	0.2527
148		Mutuntu	0.2439	0.2711	0.3001	0.273	0.3012	0.331	0.3614	0.39	0.4195	0.2186	0.2445	0.2723
149		Rubengera	0.1997	0.2209	0.2437	0.1932	0.2153	0.2392	0.3517	0.3763	0.4014	0.2863	0.3153	0.3459
150		Rugabano	0.2659	0.2924	0.3203	0.2739	0.3008	0.3292	0.4282	0.4538	0.4797	0.3009	0.33	0.3604

151		Ruganda	0.2379	0.2726	0.3103	0.2264	0.2605	0.2979	0.3323	0.3694	0.4081	0.3567	0.3951	0.4348
152		Rwankuba	0.2104	0.2347	0.261	0.2672	0.2934	0.3211	0.3246	0.3488	0.3738	0.3088	0.3365	0.3653
153		Twumba	0.2133	0.2426	0.2746	0.233	0.2622	0.2937	0.3212	0.3466	0.3729	0.1927	0.2213	0.2528
154		Gahini	0.3121	0.3454	0.3802	0.1818	0.205	0.2303	0.4217	0.4538	0.4863	0.3476	0.3743	0.4019
155		Kabare	0.1868	0.2162	0.2489	0.2715	0.3122	0.3561	0.3329	0.3677	0.404	0.2538	0.2824	0.313
156		Kabarondo	0.1904	0.2161	0.2442	0.1551	0.1779	0.2034	0.3932	0.4149	0.4369	0.2169	0.2479	0.2817
157		Mukarange	0.0938	0.1083	0.1249	0.1463	0.1635	0.1822	0.3121	0.3458	0.381	0.1749	0.1978	0.2228
158		Murama	0.2876	0.3267	0.3684	0.1319	0.1604	0.1937	0.423	0.4627	0.503	0.256	0.2939	0.3348
159	Kayonza	Murundi	0.4719	0.4947	0.5175	0.2669	0.2875	0.309	0.1645	0.1818	0.2006	0.183	0.2029	0.2244
160		Mwiri	0.1713	0.1928	0.2164	0.1125	0.1327	0.1559	0.2642	0.2984	0.335	0.2526	0.276	0.3007
161		Ndego	0.2677	0.3326	0.4046	0.2092	0.2445	0.2837	0.1788	0.2007	0.2245	0.2278	0.2716	0.3203
162		Nyamirama	0.338	0.3724	0.4082	0.2125	0.2386	0.2668	0.3881	0.4205	0.4536	0.3866	0.4205	0.4551
163		Rukara	0.1117	0.1287	0.1479	0.2089	0.2298	0.252	0.3417	0.3804	0.4207	0.2009	0.2265	0.2543
164		Ruramira	0.2557	0.2936	0.3347	0.1799	0.2109	0.2457	0.3253	0.3629	0.4022	0.1914	0.2296	0.2729
165		Rwinkwavu	0.2988	0.3462	0.3969	0.2015	0.231	0.2635	0.2572	0.282	0.3082	0.3624	0.3915	0.4214
166		Gahanga	0.2153	0.2386	0.2636	0.1523	0.1665	0.1818	0.2319	0.2544	0.2783	0.1078	0.2117	0.3737
167		Gatenga	0.261	0.284	0.3082	0.1391	0.1588	0.1807	0.2315	0.2531	0.276	0.1004	0.1219	0.1474
168		Gikondo	0.1485	0.195	0.2518	0.0646	0.0908	0.1263	0.1589	0.2104	0.2731	0.1183	0.1628	0.2199
169		Kagarama	0.1445	0.1943	0.2561	0.0686	0.0967	0.1348	0.2412	0.2814	0.3255	0.1375	0.1887	0.2532
170	Kicukiro	Kanombe	0.1771	0.1972	0.2191	0.1204	0.1372	0.156	0.2735	0.2957	0.3191	0.2462	0.2751	0.3059
171		Kicukiro	0.1646	0.2096	0.2629	0.0993	0.1369	0.1857	0.2197	0.2655	0.317	0.1042	0.147	0.2033
172		Kigarama	0.15	0.1724	0.1974	0.1167	0.1336	0.1526	0.199	0.2238	0.2508	0.1577	0.1905	0.2282
173		Masaka	0.1989	0.2197	0.242	0.0746	0.0877	0.1028	0.2507	0.272	0.2944	0.1486	0.1677	0.1887
174		Niboye	0.2135	0.2584	0.309	0.0583	0.089	0.1336	0.1983	0.237	0.2807	0.0583	0.0892	0.1342
175		Nyarugunga	0.0401	0.0523	0.0679	0.0639	0.0794	0.0982	0.2208	0.2531	0.2883	0.1242	0.1483	0.176
176		Gahara	0.1022	0.1168	0.1331	0.079	0.0924	0.1079	0.4737	0.4988	0.5238	0.2354	0.2714	0.3108
177		Gatore	0.1114	0.1294	0.1498	0.0664	0.0817	0.1001	0.2657	0.2961	0.3283	0.3172	0.3525	0.3896
178		Kigarama	0.1341	0.1538	0.1757	0.0665	0.0793	0.0943	0.2567	0.2824	0.3096	0.238	0.2629	0.2894
179		Kigina	0.0681	0.0826	0.0999	0.1463	0.166	0.1877	0.3112	0.3404	0.3708	0.3836	0.4156	0.4483
180		Kirehe	0.047	0.059	0.0739	0.0274	0.0372	0.0503	0.1641	0.1832	0.2039	0.6159	0.6488	0.6804
181	Kirehe	Mahama	0.1527	0.1657	0.1795	0.1778	0.1911	0.2051	0.3007	0.3295	0.3596	0.2961	0.3238	0.3527
182		Mpanga	0.1359	0.1545	0.1752	0.089	0.1046	0.1225	0.2811	0.3053	0.3306	0.1717	0.1914	0.2128
183		Musaza	0.1937	0.2192	0.2469	0.0982	0.1157	0.1359	0.6306	0.6669	0.7013	0.3381	0.369	0.401
184		Mushikiri	0.1591	0.1801	0.2032	0.0885	0.1054	0.1251	0.2443	0.2684	0.2939	0.2847	0.3144	0.3458
185		Nasho	0.1749	0.1977	0.2226	0.0719	0.0882	0.1077	0.2714	0.3004	0.3312	0.2279	0.2729	0.3231
186		Nyamugari	0.0779	0.0913	0.1068	0.0874	0.101	0.1164	0.3687	0.3919	0.4156	0.2542	0.2751	0.297
187		Nyarubuye	0.1463	0.1726	0.2026	0.1502	0.1751	0.203	0.2288	0.2621	0.2982	0.2643	0.2939	0.3254
188		Cyeza	0.2046	0.2329	0.2637	0.1208	0.1413	0.1646	0.3656	0.3955	0.4262	0.1795	0.2209	0.2686
189		Kabacuzi	0.3097	0.3448	0.3816	0.1082	0.128	0.1509	0.4895	0.5264	0.563	0.1918	0.2282	0.2692
190		Kibangu	0.2252	0.2625	0.3037	0.1979	0.2293	0.2639	0.3116	0.3535	0.3977	0.2528	0.2929	0.3365
191		Kiyumba	0.1193	0.1441	0.1731	0.1022	0.1258	0.1539	0.2943	0.325	0.3573	0.1585	0.191	0.2284
192		Muhanga	0.1098	0.129	0.151	0.0339	0.0444	0.0578	0.5008	0.5536	0.6052	0.3125	0.3573	0.4047
193	Muhanga	Mushishiro	0.1296	0.1537	0.1812	0.1576	0.1843	0.2143	0.4613	0.4974	0.5335	0.2687	0.3092	0.353
194		Nyabinoni	0.187	0.2225	0.2625	0.2145	0.2493	0.2876	0.5063	0.5668	0.6253	0.1623	0.2558	0.3787
195		Nyamabuye	0.2301	0.25	0.2711	0.0435	0.0531	0.0647	0.3683	0.3917	0.4156	0.37	0.403	0.4369
196		Nyarusange	0.0836	0.1001	0.1196	0.1347	0.1565	0.181	0.3362	0.3715	0.4083	0.1983	0.2332	0.2721
197		Rongi	0.1619	0.1849	0.2103	0.0912	0.109	0.1299	0.3532	0.3862	0.4202	0.3356	0.3759	0.4181
198		Rugendabari	0.0855	0.1088	0.1377	0.0698	0.0891	0.1132	0.2841	0.3198	0.3577	0.2709	0.3278	0.3903
199		Shyogwe	0.1325	0.1538	0.1778	0.1183	0.1366	0.1572	0.3502	0.3778	0.4062	0.3524	0.3933	0.4357
200		Busogo	0.1733	0.2048	0.2404	0.1729	0.1988	0.2276	0.5013	0.5316	0.5616	0.1841	0.2159	0.2514
201		Cyuve	0.3003	0.3238	0.3482	0.1486	0.1644	0.1814	0.3891	0.4114	0.4341	0.2966	0.3272	0.3594
202		Gacaca	0.2513	0.2756	0.3013	0.2798	0.3059	0.3333	0.324	0.3556	0.3885	0.3274	0.3572	0.388
203		Gashaki	0.2948	0.3326	0.3727	0.2212	0.2565	0.2953	0.2105	0.2424	0.2774	0.2459	0.2837	0.3248
204		Gataraga	0.247	0.2876	0.3319	0.1545	0.1846	0.2191	0.4397	0.4707	0.502	0.2581	0.2972	0.3396
205		Kimonyi	0.3075	0.3372	0.3684	0.271	0.3002	0.3311	0.4814	0.5154	0.5492	0.3316	0.374	0.4184
206		Kinigi	0.2303	0.2592	0.2904	0.1969	0.2215	0.2482	0.4356	0.4606	0.4858	0.3747	0.4279	0.4827
207	Musanze	Muhoza	0.2412	0.2621	0.2842	0.0534	0.0636	0.0756	0.3287	0.349	0.3699	0.2198	0.2517	0.2864
208		Muko	0.2355	0.2626	0.2917	0.2254	0.2541	0.2851	0.4727	0.5041	0.5354	0.3023	0.3376	0.3747
209		Musanze	0.2609	0.285	0.3103	0.2204	0.2397	0.2602	0.4044	0.4273	0.4505	0.3517	0.3822	0.4137
210		Nkotsi	0.2204	0.2612	0.3066	0.1554	0.1873	0.2241	0.36	0.4007	0.4428	0.1885	0.2516	0.3272
211		Nyange	0.2346	0.2631	0.2937	0.1924	0.2187	0.2475	0.2364	0.2645	0.2947	0.3218	0.3548	0.3892
212		Remera	0.2578	0.2911	0.3268	0.2496	0.2848	0.3227	0.3441	0.3755	0.4081	0.2789	0.3201	0.3644
213		Rwaza	0.2201	0.2492	0.2807	0.1269	0.1522	0.1815	0.3212	0.3505	0.3809	0.3069	0.3487	0.3928
214		Shingiro	0.1558	0.1816	0.2107	0.2535	0.2861	0.3212	0.3814	0.4152	0.4499	0.2511	0.283	0.3172
215		Gashanda	0.3672	0.404	0.4419	0.2195	0.247	0.2767	0.3023	0.338	0.3756	0.3015	0.337	0.3745
216		Jarama	0.2676	0.2928	0.3193	0.0554	0.0671	0.081	0.3763	0.4056	0.4355	0.3757	0.4049	0.4348
217		Karembo	0.034	0.0477	0.0665	0.0186	0.0281	0.0422	0.3614	0.401	0.4419	0.3599	0.3994	0.4404
218	Ngoma	Kazo	0.305	0.3322	0.3605	0.1817	0.203	0.2261	0.3644	0.3958	0.4282	0.3644	0.3959	0.4282
219		Kibungo	0.2788	0.308	0.3388	0.0708	0.0849	0.1016	0.3918	0.4201	0.4488	0.391	0.4193	0.448
220		Mugesera	0.2395	0.2645	0.2912	0.0943	0.1119	0.1324	0.2402	0.2758	0.3144	0.2395	0.2749	0.3134

221		Murama	0.3106	0.3398	0.3703	0.1671	0.1897	0.2146	0.2687	0.2959	0.3246	0.2701	0.2973	0.3261
222		Mutenderi	0.3581	0.3889	0.4207	0.1351	0.157	0.1817	0.3237	0.3573	0.3924	0.3225	0.3561	0.3912
223		Remera	0.2381	0.2626	0.2887	0.1189	0.1366	0.1565	0.3054	0.3332	0.3621	0.3055	0.3332	0.3622
224		Rukira	0.2315	0.2567	0.2836	0.0415	0.0522	0.0656	0.2354	0.2608	0.2879	0.2366	0.262	0.289
225		Rukumberi	0.1473	0.1659	0.1864	0.0537	0.0648	0.078	0.3822	0.4132	0.445	0.3833	0.4144	0.4462
226		Rurenge	0.3179	0.3435	0.3701	0.0747	0.0887	0.1049	0.3084	0.3348	0.3622	0.3084	0.3348	0.3623
227		Sake	0.3159	0.3423	0.3697	0.1577	0.1791	0.2027	0.3863	0.4216	0.4577	0.3877	0.423	0.4592
228		Zaza	0.0625	0.0774	0.0954	0.0358	0.0464	0.0598	0.3225	0.3483	0.375	0.3236	0.3494	0.376
229		Bwira	0.2293	0.2656	0.3054	0.2819	0.3143	0.3487	0.2493	0.2769	0.3064	0.3628	0.4145	0.4682
230		Gatumba	0.1374	0.1592	0.1838	0.1667	0.192	0.22	0.372	0.4028	0.4343	0.2657	0.302	0.341
231		Hindiro	0.3047	0.3376	0.3722	0.2815	0.3155	0.3516	0.2825	0.3194	0.3588	0.2283	0.2684	0.3126
232		Kabaya	0.1859	0.2066	0.2289	0.1264	0.1442	0.1641	0.3737	0.3993	0.4253	0.2583	0.2879	0.3194
233		Kageyo	0.1026	0.1233	0.1476	0.2386	0.2693	0.3023	0.6179	0.6492	0.6794	0.5585	0.5916	0.6238
234		Kavumu	0.2744	0.3023	0.3318	0.2809	0.3106	0.342	0.3309	0.3595	0.3892	0.2291	0.2627	0.2993
235	Ngororero	Matyazo	0.2016	0.2303	0.2618	0.2418	0.2693	0.2986	0.2579	0.2866	0.3172	0.2479	0.2821	0.3189
236		Muhanda	0.3203	0.3596	0.4007	0.2093	0.2372	0.2675	0.4385	0.4721	0.506	0.5816	0.6243	0.6652
237		Muhororo	0.1878	0.2143	0.2434	0.1853	0.2139	0.2456	0.2778	0.3083	0.3405	0.2463	0.2839	0.3247
238		Ndaro	0.2165	0.2454	0.2769	0.2689	0.2995	0.332	0.7295	0.756	0.7806	0.6694	0.7062	0.7405
239		Ngororero	0.1671	0.1886	0.2121	0.1272	0.1448	0.1645	0.4267	0.451	0.4755	0.3787	0.4066	0.4352
240		Nyange	0.1518	0.1768	0.205	0.1847	0.2111	0.2401	0.4166	0.4475	0.4788	0.217	0.27	0.3305
241		Sovu	0.321	0.3568	0.3942	0.3501	0.3815	0.4139	0.4296	0.4627	0.4961	0.559	0.5959	0.6317
242		Bigogwe	0.2872	0.3171	0.3486	0.3321	0.3589	0.3867	0.2747	0.3011	0.3288	0.3157	0.3429	0.3711
243		Jenda	0.4026	0.4265	0.4508	0.3354	0.3591	0.3836	0.3603	0.3881	0.4166	0.4476	0.4775	0.5075
244		Jomba	0.2215	0.2595	0.3016	0.3656	0.4032	0.442	0.1486	0.1714	0.1968	0.3171	0.3626	0.4106
245		Kabatwa	0.329	0.3667	0.4062	0.2881	0.3205	0.3548	0.4013	0.4378	0.475	0.3531	0.3939	0.4362
246		Karago	0.2754	0.3051	0.3364	0.3212	0.3501	0.3802	0.3379	0.3686	0.4004	0.2834	0.3166	0.3517
247	Nyabihu	Kintobo	0.1947	0.2335	0.2774	0.2699	0.3067	0.3461	0.3403	0.386	0.4337	0.2736	0.3197	0.3697
248		Mukamira	0.3167	0.3467	0.378	0.1608	0.1809	0.2029	0.3475	0.3826	0.419	0.3069	0.3364	0.3672
249		Muringa	0.3762	0.4135	0.4519	0.3425	0.3748	0.4082	0.2648	0.298	0.3336	0.3664	0.4203	0.4761
250		Rambura	0.1666	0.1903	0.2164	0.2594	0.285	0.3121	0.2028	0.2273	0.2537	0.1759	0.2014	0.2295
251		Rugera	0.3475	0.382	0.4178	0.2436	0.269	0.2961	0.3344	0.364	0.3946	0.2883	0.32	0.3535
252		Rurembo	0.4006	0.4362	0.4724	0.2513	0.2795	0.3096	0.3262	0.3642	0.404	0.2708	0.3075	0.3467
253		Shyira	0.1855	0.2174	0.253	0.2673	0.3012	0.3375	0.2838	0.3197	0.3578	0.234	0.2629	0.2939
254		Gatunda	0.3804	0.4065	0.4332	0.2621	0.2887	0.3169	0.4164	0.4428	0.4695	0.3907	0.4168	0.4434
255		Karama	0.2948	0.3227	0.3519	0.2082	0.2348	0.2637	0.2408	0.2669	0.2947	0.2279	0.2535	0.2809
256		Karangazi	0.1356	0.1489	0.1633	0.1042	0.118	0.1333	0.3613	0.3784	0.3958	0.3513	0.3682	0.3855
257		Katabagemu	0.2895	0.3122	0.3358	0.2063	0.2262	0.2474	0.2231	0.2442	0.2667	0.2192	0.2403	0.2626
258		Kiyombe	0.3108	0.3449	0.3807	0.2098	0.2407	0.2745	0.4662	0.5001	0.5341	0.4531	0.487	0.521
259		Matimba	0.2351	0.2597	0.2858	0.1097	0.1292	0.1517	0.3245	0.3529	0.3823	0.3125	0.3406	0.3698
260	Nyagatare	Mimuri	0.3277	0.3535	0.3802	0.2616	0.286	0.3117	0.4069	0.4329	0.4594	0.3925	0.4183	0.4446
261		Mukama	0.3241	0.354	0.3852	0.2542	0.282	0.3116	0.355	0.3829	0.4116	0.3458	0.3736	0.4022
262		Musheri	0.1945	0.219	0.2457	0.2	0.2243	0.2506	0.5112	0.5305	0.5497	0.5046	0.5239	0.5431
263		Nyagatare	0.3148	0.3352	0.3562	0.1683	0.182	0.1966	0.366	0.3858	0.4059	0.3534	0.373	0.393
264		Rukomo	0.4	0.4257	0.4518	0.219	0.2402	0.2629	0.3351	0.3567	0.3789	0.3274	0.3489	0.371
265		Rwempasha	0.3226	0.356	0.3908	0.1759	0.1994	0.2252	0.0909	0.1103	0.1332	0.0843	0.103	0.1253
266		Rwimiyaga	0.2082	0.2297	0.2527	0.2383	0.2662	0.2961	0.4727	0.4967	0.5207	0.4638	0.4878	0.5118
267		Tabagwe	0.2493	0.2715	0.295	0.1797	0.1984	0.2185	0.4916	0.513	0.5344	0.4856	0.5071	0.5285
268		Buruhukiro	0.3522	0.3817	0.4121	0.0875	0.1047	0.1249	0.3653	0.3991	0.4339	0.326	0.3582	0.3917
269		Cyanika	0.2175	0.2474	0.28	0.136	0.1572	0.1811	0.2712	0.299	0.3283	0.2699	0.2989	0.3296
270		Gasaka	0.2354	0.2605	0.2873	0.1335	0.1528	0.1743	0.3058	0.3331	0.3616	0.1812	0.2035	0.2276
271		Gatare	0.3119	0.3485	0.3871	0.2428	0.2774	0.3149	0.4363	0.4799	0.5238	0.3578	0.401	0.4459
272		Kaduha	0.2705	0.3049	0.3417	0.2158	0.2432	0.273	0.3628	0.396	0.4302	0.5209	0.5575	0.5935
273		Kamegeri	0.1077	0.1345	0.1667	0.1161	0.1441	0.1774	0.3098	0.3524	0.3975	0.2789	0.3261	0.3771
274		Kibirizi	0.3207	0.3616	0.4047	0.1327	0.1572	0.1852	0.3455	0.3826	0.421	0.2841	0.3216	0.3615
275	Nyamagabe	Kibumbwe	0.0465	0.063	0.0849	0.177	0.2094	0.2459	0.2867	0.3401	0.3979	0.2901	0.3325	0.3777
276		Kitabi	0.1057	0.1256	0.1487	0.0859	0.1019	0.1205	0.3564	0.3867	0.4178	0.4298	0.46	0.4904
277		Mbazi	0.1387	0.1717	0.2107	0.1508	0.1831	0.2204	0.2435	0.2931	0.3481	0.2112	0.255	0.3044
278		Mugano	0.1309	0.1582	0.1899	0.2439	0.276	0.3105	0.3123	0.3481	0.3857	0.2271	0.2627	0.3018
279		Musange	0.3386	0.376	0.415	0.1175	0.143	0.1729	0.3907	0.429	0.4681	0.3082	0.3547	0.4041
280		Musebeya	0.2858	0.3176	0.3513	0.1301	0.1542	0.1819	0.2821	0.3141	0.3478	0.344	0.3804	0.4182
281		Mushubi	0.2379	0.275	0.3156	0.0533	0.0717	0.0957	0.3107	0.3535	0.3987	0.2941	0.3283	0.3645
282		Nkomane	0.254	0.289	0.3268	0.174	0.2004	0.2296	0.3471	0.384	0.4223	0.3538	0.4152	0.4794
283		Tare	0.3528	0.3845	0.4173	0.2583	0.2862	0.3158	0.3351	0.3642	0.3944	0.3523	0.3891	0.4273
284		Uwinkingi	0.1576	0.1822	0.2096	0.3477	0.3792	0.4118	0.3545	0.3852	0.4169	0.2792	0.3159	0.3551
285		Bushekeri	0.2662	0.2988	0.3336	0.2512	0.2847	0.3207	0.4155	0.442	0.4689	0.3233	0.3505	0.3787
286		Bushenge	0.1974	0.2285	0.2628	0.1562	0.1844	0.2165	0.3291	0.3584	0.3888	0.2206	0.2556	0.294
287	Nyamasheke	Cyato	0.6451	0.674	0.7016	0.3481	0.3789	0.4108	0.4169	0.442	0.4673	0.3729	0.4075	0.443
288		Gihombo	0.3358	0.3674	0.4001	0.2627	0.2942	0.3279	0.45	0.4792	0.5086	0.305	0.3518	0.4017
289		Kagano	0.2633	0.292	0.3225	0.1092	0.1272	0.1476	0.5717	0.599	0.6258	0.3118	0.338	0.3651
290		Kanjongo	0.208	0.2368	0.2683	0.2011	0.2275	0.2562	0.3226	0.3458	0.3698	0.2203	0.2496	0.2813

291		Karambi	0.3562	0.3892	0.4232	0.2576	0.2866	0.3175	0.4131	0.4416	0.4704	0.3606	0.3998	0.4402
292		Karengera	0.2264	0.2523	0.2801	0.1482	0.1713	0.197	0.2564	0.279	0.3027	0.2955	0.3283	0.3628
293		Kirimbi	0.3142	0.3519	0.3916	0.286	0.3174	0.3506	0.4927	0.5235	0.5542	0.337	0.3906	0.447
294		Macuba	0.3602	0.3903	0.4213	0.1883	0.2142	0.2426	0.4399	0.4688	0.498	0.141	0.1778	0.2218
295		Mahembe	0.3174	0.3583	0.4015	0.2009	0.2353	0.2737	0.3647	0.411	0.4589	0.2722	0.3072	0.3446
296		Nyabitekeri	0.2423	0.2731	0.3063	0.1171	0.1396	0.1656	0.5514	0.5814	0.6108	0.2045	0.2441	0.2885
297		Rangiro	0.3073	0.3419	0.3784	0.2887	0.3254	0.3642	0.5517	0.5911	0.6294	0.3746	0.4198	0.4665
298		Ruharambuga	0.1382	0.1658	0.1977	0.2003	0.2304	0.2635	0.4599	0.4914	0.523	0.4633	0.4947	0.5261
299		Shangi	0.1872	0.2113	0.2375	0.1304	0.1527	0.178	0.3906	0.4193	0.4486	0.2455	0.2759	0.3085
300		Busasamana	0.1973	0.2168	0.2376	0.0799	0.1013	0.1277	0.1504	0.1718	0.1956	0.1728	0.1959	0.2213
301		Busoro	0.274	0.2977	0.3226	0.1544	0.1832	0.216	0.284	0.3108	0.339	0.1371	0.165	0.1974
302		Cyabakamyi	0.3344	0.3704	0.4079	0.0844	0.116	0.1572	0.1769	0.2016	0.2288	0.307	0.3497	0.3949
303		Kibilizi	0.2266	0.2499	0.2747	0.0389	0.0511	0.067	0.1918	0.2159	0.2422	0.2015	0.2252	0.2508
304	Nyanza	Kigoma	0.2539	0.2775	0.3023	0.1088	0.1299	0.1543	0.2137	0.2419	0.2724	0.2567	0.3036	0.3549
305		Mukingo	0.1769	0.1997	0.2245	0.1203	0.1432	0.1696	0.2424	0.2703	0.3001	0.1967	0.2322	0.2719
306		Muyira	0.2858	0.3091	0.3334	0.037	0.0537	0.0773	0.3889	0.4213	0.4544	0.2094	0.2434	0.281
307		Ntyazo	0.1586	0.1795	0.2026	0.0705	0.0886	0.1107	0.3205	0.364	0.41	0.2446	0.2866	0.3326
308		Nyagisozi	0.2359	0.2625	0.291	0.0667	0.0844	0.1062	0.2466	0.2774	0.3105	0.2392	0.2784	0.3213
309		Rwabicuma	0.1795	0.2079	0.2394	0.071	0.0919	0.118	0.2058	0.2423	0.2829	0.1684	0.1982	0.2318
310		Gitega	0.123	0.1445	0.169	0.1023	0.1232	0.1476	0.2396	0.2714	0.3056	0.1284	0.1581	0.1933
311		Kanyinya	0.103	0.1244	0.1496	0.0289	0.0385	0.0512	0.3486	0.3815	0.4157	0.2982	0.3268	0.3568
312		Kigali	0.1387	0.1604	0.1847	0.0525	0.0631	0.0757	0.102	0.1192	0.1388	0.3277	0.3585	0.3906
313		Kimisagara	0.1481	0.1718	0.1986	0.1239	0.1435	0.1656	0.2079	0.2307	0.2553	0.1897	0.2217	0.2574
314		Mageragere	0.1075	0.125	0.1449	0.0664	0.0779	0.0913	0.6253	0.6481	0.6703	0.2502	0.2834	0.3192
315	Nyarugenge	Muhima	0.0718	0.0967	0.129	0.0212	0.0314	0.0461	0.0702	0.0913	0.1179	0.1737	0.2173	0.2684
316		Nyakabanda	0.022	0.033	0.049	0.0359	0.0495	0.0678	0.0918	0.2145	0.4247	0.1842	0.2843	0.4115
317		Nyamirambo	0.0758	0.0903	0.1072	0.1013	0.1163	0.1331	0.3147	0.3351	0.3562	0.3304	0.3545	0.3794
318		Nyarugenge	0.0474	0.071	0.105	0.0335	0.0519	0.0795	0.1803	0.2268	0.2812	0.1134	0.1693	0.2451
319		Rwezamenyo	0.0491	0.0744	0.1112	0.0288	0.0462	0.0731	0.0807	0.1943	0.3984	0.2446	0.2903	0.3408
320		Busanze	0.0592	0.0726	0.0889	0.2192	0.242	0.2664	0.3204	0.3462	0.3729	0.3056	0.3431	0.3827
321		Cyahinda	0.1389	0.1621	0.1883	0.215	0.2421	0.2714	0.2239	0.2507	0.2796	0.2788	0.3107	0.3445
322		Kibeho	0.1622	0.1845	0.2091	0.2402	0.2681	0.2979	0.1588	0.181	0.2055	0.3163	0.3455	0.3758
323		Kivu	0.3184	0.3534	0.39	0.3298	0.3639	0.3995	0.3623	0.3962	0.4311	0.4133	0.452	0.4914
324		Mata	0.1913	0.2217	0.2555	0.254	0.286	0.3204	0.4383	0.4779	0.5178	0.3712	0.4118	0.4536
325		Muganza	0.1974	0.2241	0.2532	0.2499	0.2808	0.314	0.2731	0.3005	0.3293	0.2745	0.3084	0.3445
326	Nyaruguru	Munini	0.2058	0.2356	0.2684	0.2193	0.2487	0.2807	0.1922	0.2217	0.2543	0.251	0.2857	0.3232
327		Ngera	0.2174	0.2469	0.2791	0.2063	0.2309	0.2576	0.3001	0.3271	0.3553	0.2531	0.2874	0.3243
328		Ngoma	0.2624	0.2926	0.3247	0.3839	0.4195	0.4559	0.4219	0.4543	0.4871	0.2085	0.2363	0.2666
329		Nyabimata	0.2703	0.3063	0.3448	0.2895	0.3212	0.3547	0.2978	0.3257	0.355	0.3094	0.3522	0.3975
330		Nyagisozi	0.0652	0.0834	0.1061	0.0962	0.115	0.137	0.2535	0.2833	0.3152	0.3348	0.3707	0.4081
331		Ruheru	0.1843	0.2068	0.2313	0.2975	0.3246	0.3529	0.3786	0.4067	0.4354	0.3538	0.3921	0.4317
332		Ruramba	0.2273	0.2602	0.296	0.3437	0.3766	0.4107	0.2727	0.3021	0.3333	0.2709	0.3298	0.3945
333		Rusenge	0.2314	0.2575	0.2854	0.2672	0.2947	0.3238	0.4473	0.4777	0.5082	0.3377	0.3681	0.3996
334		Bugeshi	0.2377	0.261	0.2856	0.4404	0.4796	0.519	0.3537	0.3986	0.4453	0.4679	0.497	0.5261
335		Busasamana	0.105	0.1195	0.1358	0.1551	0.176	0.199	0.2964	0.3197	0.3439	0.3263	0.3491	0.3725
336		Cyanzarwe	0.104	0.1219	0.1423	0.3352	0.3633	0.3923	0.5528	0.5794	0.6055	0.4933	0.5184	0.5434
337		Gisenyi	0.1304	0.1491	0.17	0.2263	0.2481	0.2712	0.1307	0.147	0.1649	0.3986	0.4394	0.4811
338		Kanama	0.2721	0.2977	0.3247	0.385	0.414	0.4436	0.4461	0.4756	0.5051	0.5173	0.5421	0.5666
339		Kanzenze	0.2391	0.2709	0.3052	0.3307	0.3625	0.3956	0.3415	0.3797	0.4193	0.4008	0.4374	0.4748
340	Rubavu	Mudende	0.2881	0.3173	0.348	0.3454	0.3737	0.403	0.4349	0.4639	0.4931	0.384	0.4175	0.4518
341		Nyakiriba	0.0806	0.0958	0.1135	0.232	0.2568	0.2832	0.3408	0.3769	0.4144	0.4674	0.4939	0.5203
342		Nyamyumba	0.2369	0.2617	0.2881	0.3133	0.3379	0.3634	0.5099	0.5306	0.5513	0.5165	0.5351	0.5536
343		Nyundo	0.2366	0.2588	0.2824	0.4391	0.4673	0.4958	0.5512	0.5758	0.6	0.4846	0.5099	0.5353
344		Rubavu	0.2203	0.2401	0.2611	0.2928	0.3116	0.3311	0.3995	0.4209	0.4426	0.3347	0.359	0.3841
345		Rugerero	0.1375	0.1531	0.1702	0.1651	0.1814	0.1989	0.2226	0.2374	0.2528	0.4164	0.4421	0.4681
346		Bweramana	0.1414	0.1659	0.1936	0.1846	0.2081	0.2336	0.2851	0.3095	0.335	0.2841	0.3084	0.3338
347		Byimana	0.267	0.2992	0.3334	0.2171	0.2411	0.2667	0.3769	0.4014	0.4265	0.3765	0.401	0.426
348		Kabagali	0.2621	0.2941	0.3284	0.1572	0.1805	0.2064	0.3468	0.379	0.4122	0.3472	0.3793	0.4126
349		Kinazi	0.1868	0.2049	0.2242	0.1964	0.2163	0.2377	0.3673	0.3935	0.4203	0.3673	0.3934	0.4202
350	Ruhango	Kinihira	0.253	0.2803	0.3093	0.2018	0.2299	0.2607	0.3069	0.3362	0.3669	0.306	0.3353	0.3659
351		Mbuye	0.2482	0.2713	0.2957	0.1856	0.2076	0.2314	0.4288	0.4504	0.4722	0.4278	0.4494	0.4712
352		Mwendo	0.234	0.2621	0.2923	0.1649	0.1898	0.2174	0.2929	0.3224	0.3535	0.2927	0.3222	0.3532
353		Ntongwe	0.2836	0.3078	0.3331	0.2065	0.2293	0.2539	0.4307	0.4568	0.4831	0.4302	0.4562	0.4824
354		Ruhango	0.2042	0.2201	0.237	0.1885	0.2047	0.2219	0.4292	0.4469	0.4648	0.4294	0.4472	0.4651
355	Rulindo	Base	0.2846	0.3308	0.3804	0.2318	0.2645	0.3	0.1615	0.1864	0.214	0.1766	0.3597	0.5953
356		Burega	0.3319	0.3765	0.4232	0.2986	0.3419	0.3881	0.6131	0.6507	0.6866	0.4154	0.4709	0.5273

357		Bushoki	0.2902	0.3212	0.3538	0.2137	0.2442	0.2775	0.3112	0.3433	0.3769	0.2846	0.3292	0.3772
358		Buyoga	0.2223	0.253	0.2864	0.1977	0.225	0.255	0.397	0.4276	0.4589	0.3242	0.3652	0.4082
359		Cyinzuzi	0.3971	0.4389	0.4816	0.193	0.2252	0.2612	0.2057	0.2839	0.3777	0.4834	0.5315	0.579
360		Cyungo	0.3187	0.3627	0.4092	0.2398	0.2769	0.3174	0.3709	0.4142	0.4587	0.3117	0.3586	0.4084
361		Kinihira	0.2835	0.3196	0.3579	0.2723	0.3152	0.3614	0.2627	0.2958	0.3312	0.1672	0.201	0.2396
362		Kisaro	0.3149	0.3505	0.3878	0.2729	0.3052	0.3395	0.4328	0.4678	0.503	0.2484	0.2846	0.3237
363		Masoro	0.1991	0.2305	0.2652	0.1532	0.179	0.2082	0.3719	0.4024	0.4337	0.3118	0.3513	0.3929
364		Mbogo	0.2155	0.2528	0.2942	0.2071	0.2416	0.2799	0.4098	0.4457	0.4822	0.2771	0.3167	0.3592
365		Murambi	0.1997	0.2316	0.2669	0.1498	0.1721	0.197	0.521	0.5597	0.5977	0.3887	0.4322	0.4767
366		Ngoma	0.1071	0.14	0.181	0.146	0.1812	0.2226	0.3575	0.4345	0.5149	0.3584	0.412	0.4677
367		Ntarabana	0.1955	0.2297	0.268	0.2722	0.3019	0.3333	0.3645	0.3976	0.4316	0.3131	0.3712	0.4333
368		Rukozo	0.3011	0.3428	0.3871	0.2521	0.2888	0.3284	0.4481	0.4905	0.5331	0.2984	0.3516	0.4089
369		Rusiga	0.2865	0.3404	0.3987	0.1584	0.1954	0.2386	0.3434	0.3902	0.4392	0.2814	0.3325	0.388
370		Shyorongi	0.2404	0.2687	0.2992	0.1689	0.1898	0.2127	0.2182	0.2442	0.2722	0.2339	0.2631	0.2945
371		Tumba	0.246	0.292	0.3427	0.1619	0.1927	0.2277	0.5075	0.5386	0.5694	0.2791	0.3284	0.3819
372		Bugarama	0.4691	0.5081	0.547	0.1937	0.2128	0.2333	0.5703	0.6099	0.648	0.5676	0.6075	0.646
373		Butare	0.0735	0.0911	0.1123	0.209	0.2347	0.2624	0.4621	0.4907	0.5193	0.4613	0.4899	0.5185
374		Bweyeye	0.4421	0.5003	0.5586	0.2643	0.3038	0.3463	0.517	0.5569	0.596	0.5089	0.5488	0.588
375		Gashonga	0.322	0.351	0.3813	0.1931	0.2208	0.2512	0.3693	0.4031	0.4378	0.3396	0.3728	0.4071
376		Giheke	0.1714	0.2053	0.2439	0.1425	0.173	0.2085	0.275	0.3219	0.3726	0.2725	0.3193	0.3699
377		Gihundwe	0.2173	0.2418	0.268	0.2161	0.2417	0.2692	0.2759	0.3113	0.3491	0.2744	0.3098	0.3475
378		Gikundamvura	0.4228	0.4697	0.5172	0.1076	0.1305	0.1574	0.3979	0.4339	0.4707	0.3457	0.3807	0.417
379		Gitambi	0.3018	0.3299	0.3592	0.1332	0.1543	0.178	0.4245	0.4592	0.4943	0.4119	0.4465	0.4816
380	Rusizi	Kamembe	0.1612	0.1911	0.225	0.1519	0.1745	0.1996	0.2469	0.2765	0.3081	0.2423	0.2717	0.3032
381		Muganza	0.2624	0.2905	0.3203	0.2351	0.2604	0.2873	0.3431	0.3748	0.4076	0.3285	0.3599	0.3925
382		Mururu	0.2194	0.2431	0.2684	0.1918	0.2161	0.2425	0.2479	0.3225	0.4073	0.2411	0.3149	0.3993
383		Nkanka	0.1267	0.1593	0.1982	0.1264	0.1554	0.1895	0.2144	0.2516	0.2929	0.2125	0.2497	0.2909
384		Nkombo	0.1744	0.2018	0.2322	0.1738	0.2089	0.2489	0.3608	0.4117	0.4646	0.3582	0.4091	0.4619
385		Nkungu	0.2555	0.2877	0.3223	0.1051	0.1244	0.1467	0.3863	0.4258	0.4663	0.3497	0.3884	0.4286
386		Nyakabuye	0.3679	0.3953	0.4234	0.1176	0.1352	0.1549	0.3926	0.4283	0.4647	0.3764	0.4118	0.4481
387		Nyakarenzo	0.2479	0.2844	0.324	0.1953	0.2258	0.2595	0.3019	0.3396	0.3794	0.2853	0.3223	0.3618
388		Nzahaha	0.2349	0.2608	0.2884	0.1964	0.2205	0.2466	0.2827	0.3094	0.3375	0.2819	0.3086	0.3366
389		Rwimbogo	0.3604	0.3904	0.4213	0.1492	0.1747	0.2035	0.2811	0.3087	0.3377	0.266	0.2931	0.3218
390		Boneza	0.2831	0.3216	0.3627	0.2171	0.246	0.2773	0.4054	0.4356	0.4663	0.1704	0.3633	0.613
391		Gihango	0.2505	0.2817	0.3152	0.2308	0.2585	0.2884	0.2285	0.2558	0.2853	0.1982	0.2684	0.3526
392		Kigeyo	0.2305	0.2611	0.2942	0.2466	0.2755	0.3065	0.3253	0.3603	0.3969	0.2606	0.3098	0.3637
393		Kivumu	0.258	0.2873	0.3186	0.1932	0.2174	0.2438	0.3876	0.4122	0.4372	0.2765	0.3076	0.3404
394		Manihira	0.4245	0.4632	0.5023	0.3948	0.4326	0.4712	0.4074	0.4396	0.4723	0.47	0.5194	0.5685
395		Mukura	0.3596	0.3888	0.4187	0.1519	0.1724	0.195	0.5433	0.5687	0.5936	0.5411	0.5663	0.5913
396	Rutsiro	Murunda	0.3614	0.3933	0.4262	0.4016	0.4362	0.4714	0.496	0.529	0.5618	0.2836	0.3382	0.3975
397		Musasa	0.2076	0.2492	0.296	0.2017	0.2269	0.2543	0.2813	0.309	0.338	0.2733	0.3248	0.3809
398		Mushonyi	0.2284	0.2615	0.2976	0.201	0.2307	0.2633	0.4905	0.525	0.5593	0.4956	0.5173	0.5389
399		Mushubati	0.2491	0.2766	0.3059	0.1319	0.1547	0.1807	0.5652	0.5962	0.6264	0.3745	0.4063	0.4389
400		Nyabirasi	0.3436	0.3687	0.3946	0.2526	0.2772	0.3032	0.4468	0.4745	0.5025	0.4684	0.506	0.5436
401		Ruhango	0.3776	0.4057	0.4344	0.3634	0.3927	0.4229	0.385	0.4078	0.4311	0.2759	0.3624	0.4589
402		Rusebeya	0.4153	0.4466	0.4783	0.335	0.364	0.394	0.422	0.4562	0.4909	0.5142	0.5509	0.5871
403		Fumbwe	0.2972	0.3207	0.3452	0.0349	0.0444	0.0563	0.2438	0.2697	0.2973	0.2356	0.2612	0.2886
404		Gahengeri	0.217	0.2421	0.269	0.1883	0.2123	0.2383	0.2014	0.2254	0.2513	0.1989	0.2227	0.2485
405		Gishali	0.255	0.2823	0.3113	0.0781	0.0937	0.112	0.2344	0.2625	0.2928	0.229	0.2569	0.287
406		Karenge	0.1257	0.1463	0.1697	0.12	0.1386	0.1596	0.2945	0.3214	0.3497	0.2875	0.3142	0.3423
407		Kigabiro	0.1775	0.1957	0.2153	0.1002	0.115	0.1317	0.1763	0.1953	0.2159	0.1708	0.1895	0.2099
408		Muhazi	0.142	0.1625	0.1854	0.1301	0.1483	0.1685	0.1757	0.1972	0.2206	0.1742	0.1956	0.219
409	Rwamagana	Munyaga	0.1542	0.1797	0.2084	0.1101	0.1319	0.1572	0.2476	0.2801	0.3151	0.2421	0.2743	0.3091
410		Munyiginya	0.0603	0.0768	0.0975	0.0787	0.0952	0.1147	0.3589	0.3966	0.4356	0.3434	0.3808	0.4196
411		Musha	0.1342	0.1571	0.1831	0.0281	0.0375	0.0499	0.5874	0.6202	0.6519	0.5759	0.6088	0.6407
412		Muyumbu	0.1373	0.1538	0.1718	0.134	0.1489	0.165	0.2206	0.2422	0.2653	0.2165	0.238	0.261
413		Mwulire	0.0991	0.1164	0.1364	0.0878	0.1035	0.1217	0.1512	0.1738	0.1988	0.1471	0.1693	0.1941
414		Nyakaliro	0.1916	0.2142	0.2386	0.1048	0.1201	0.1374	0.2104	0.2363	0.2643	0.2034	0.229	0.2568
415		Nzige	0.1648	0.1916	0.2215	0.1408	0.1651	0.1927	0.3302	0.3704	0.4124	0.3212	0.3611	0.403
416		Rubona	0.2108	0.2351	0.2613	0.0689	0.0848	0.1041	0.2899	0.3168	0.345	0.2793	0.3059	0.3339
